# Real‐world evaluation of interconsensus agreement of risk of bias tools: A case study using risk of bias in nonrandomized studies‐of interventions (ROBINS‐I)

**DOI:** 10.1002/cesm.12094

**Published:** 2024-06-26

**Authors:** Samer Saadi, Bashar Hasan, Adel Kanaan, Mohamed Abusalih, Zin Tarakji, Mustafa Sadek, Ayla Shamsi Basha, Mohammed Firwana, Zhen Wang, M. Hassan Murad

**Affiliations:** ^1^ Evidence‐Based Practice Center Mayo Clinic Rochester Minnesota USA; ^2^ Robert D. and Patricia E. Kern Center for the Science of Health Care Delivery Mayo Clinic Rochester Minnesota USA

**Keywords:** interconsensus agreement, interrater reliability, methodological quality, risk of bias, ROBINS‐I, systematic reviews

## Abstract

**Background:**

Risk of bias (RoB) tools are critical in systematic reviews and affect subsequent decision‐making. RoB tools should have adequate interrater reliability and interconsensus agreement. We present an approach of post hoc evaluation of RoB tools using duplicated studies that overlap systematic reviews.

**Methods:**

Using a back‐citation approach, we identified systematic reviews that used the Risk Of Bias In Nonrandomized Studies‐of Interventions (ROBINS‐I) tool and retrieved all the included primary studies. We selected studies that were appraised by more than one systematic review and calculated observed agreement and unweighted kappa comparing the different systematic reviews' assessments.

**Results:**

We identified 903 systematic reviews that used the tool with 51,676 cited references, from which we eventually analyzed 171 duplicated studies assessed using ROBINS‐I by different systematic reviewers. The observed agreement on ROBINS‐I domains ranged from 54.9% (missing data domain) to 70.3% (deviations from intended interventions domain), and was 63.0% for overall RoB assessment of the study. Kappa coefficient ranged from 0.131 (measurement of outcome domain) to 0.396 (domains of confounding and deviations from intended interventions), and was 0.404 for overall RoB assessment of the study.

**Conclusion:**

A post hoc evaluation of RoB tools is feasible by focusing on duplicated studies that overlap systematic review. ROBINS‐I assessments demonstrated considerable variation in interconsensus agreement among various systematic reviewes that assessed the same study and outcome, suggesting the need for more intensive upfront work to calibrate systematic reviewers on how to identify context‐specific information and agree on how to judge it.

## 1 INTRODUCTION

The certainty of evidence derived from systematic reviews and meta‐analyses is fundamentally linked to the risk of bias (RoB) of included studies [1]. RoB is usually assessed using dedicated tools based on the original studies design. The validity and reliability of such tools are crucial to RoB assessment and subsequent certainty of evidence grading and decision‐making. RoB tools are usually developed via consensus of experts in methodology and undergo initial testing, feedback and modification. However, rigorous evaluation of validity and reliability is often not done. For example, a comparison of RoB assessed using the Newcastle‐Ottawa Scale between systematic reviewers and the authors of the original cohort studies showed interrater reliability by item that ranged from slight (Kappa of 0.15) to poor (Kappa of −0.06) [2]. In a different sample of studies, the Newcastle‐Ottawa Scale and the Risk of Bias In Nonrandomized Studies‐of Interventions (ROBINS‐I) were found to have higher interrate reliability (Kappa of 0.52) [3]. This suggests that the reliability of these tools is highly sensitive to the sample of studies, the topic, and likely the systematic reviewers using the tools. Thus, the original testing of tools is likely inadequate most of the time. Evaluation by the Agency for Healthcare Research and Quality Evidence‐Based Practice Program highlights this challenge [4].

Considering the difficulty of doing all this testing before the tools are released and used for RoB evaluation, post hoc evaluation of interrater reliability is needed. In this meta‐epidemiologic study, we apply a novel and pragmatic way to do this assessment by depending on studies that have been evaluated in multiple systematic reviews and a back‐citation method that allows a fast and real‐world evaluation of the use of ROBINS‐I, the contemporary tool from the Cochrane Collaboration for RoB assessment of nonrandomized studies [5].

## 2 MATERIALS AND METHODS

This study is reported according to guidelines for reporting meta‐epidemiological methodology research [6].

### 2.1 Search strategy

We searched in Scopus database for systematic reviews that cited the main publication of the ROBINS‐I tool [5]. Using a back‐citation function in Scopus, we identified all systematic reviews that have cited the tool publication. To effectively handle the extensive list of SRs identified, we focused our search scope specifically on SRs related to the top five inpatient diagnoses: septicemia, heart failure, osteoarthritis, pneumonia, and diabetes mellitus [7]. This approach increases the chance of including overlapping studies in multiple SRs. We extracted the citations of primary studies included in all systematic reviews. These citations were downloaded in RIS format to ensure compatibility with the citation management software EndNote. Once imported into EndNote, each set of references was labeled according to the review from which they originated, facilitating reference management and matching. The next step, we identified primary studies that have been included in more than one review (duplicated studies).

### 2.2 Study selection and data extraction

A subsequent title and abstract screening was performed on these duplicated studies to include nonrandomized studies potentially assessed using the ROBINS‐I tool. Full text screening of the systematic reviews that included duplicated studies was done by two authors independently to confirm two aspects: first, that the duplicated studies were indeed included in the review, and second, that the ROBINS‐I tool was used for assessment. As this tool was developed to rate RoB in studies by outcomes (i.e., the ROB ratings can vary by outcomes within a study), we included the ROB ratings that corresponded to matching outcomes. After confirmation, we extracted RoB assessments of the duplicated studies from their respective reviews.

### 2.3 ROBINS‐I tool

The tool focuses on evaluating RoB in nonrandomized studies that compare the health effects of two or more interventions [5]. An adaptation called ROBINS‐E focuses on nonrandomized studies of exposure with similar domains [8]. The tool views each study as if it mimicked a hypothetical pragmatic randomized trial and gauges how bias can affect such trial using signaling question that address seven domains: confounding, selection of participants, classification of the interventions, deviations from intended interventions, missing data, measurement of outcomes, and selection of the reported result. The first two domains address baseline issues (i.e., before the start of the intervention) and the last four domains address issues that occurred after the start of the intervention. RoB is rated as low, moderate, serious, critical or no information.

### 2.4 Interconsensus agreement

Since RoB reported in each published Cochrane systematic review was likely a result of consensus between two reviewers, the agreement measures in our study should be viewed as interconsensus agreements. We calculated the observed agreement and unweighted kappa coefficient as measures of agreement (all levels of disagreement between raters were equally weighted). We also calculated the agreement coefficient by Gwet which is less susceptible to agreement categories with extreme distribution and high agreement by chance [9, 10, 11].

We used the [irr] package in R software [12, 13].

The approach is summarized in Table 1.

**Table 1:** Proposed approach for post hoc evaluation of interrater reliability of risk of bias assessment tools.

1. Identify landmark publications of the risk of bias tool being evaluated. 2. Identify all the systematic reviews that cited the tool publications. 3. Identify primary studies that were included in more than one systematic review (duplicated studies). 4. Extract risk of bias assessments for a specific outcome from duplicated studies. 5. Statistically quantify the interrater reliability of individual items and overall risk of bias assessment.

## 3 RESULTS

**Figure 1:**
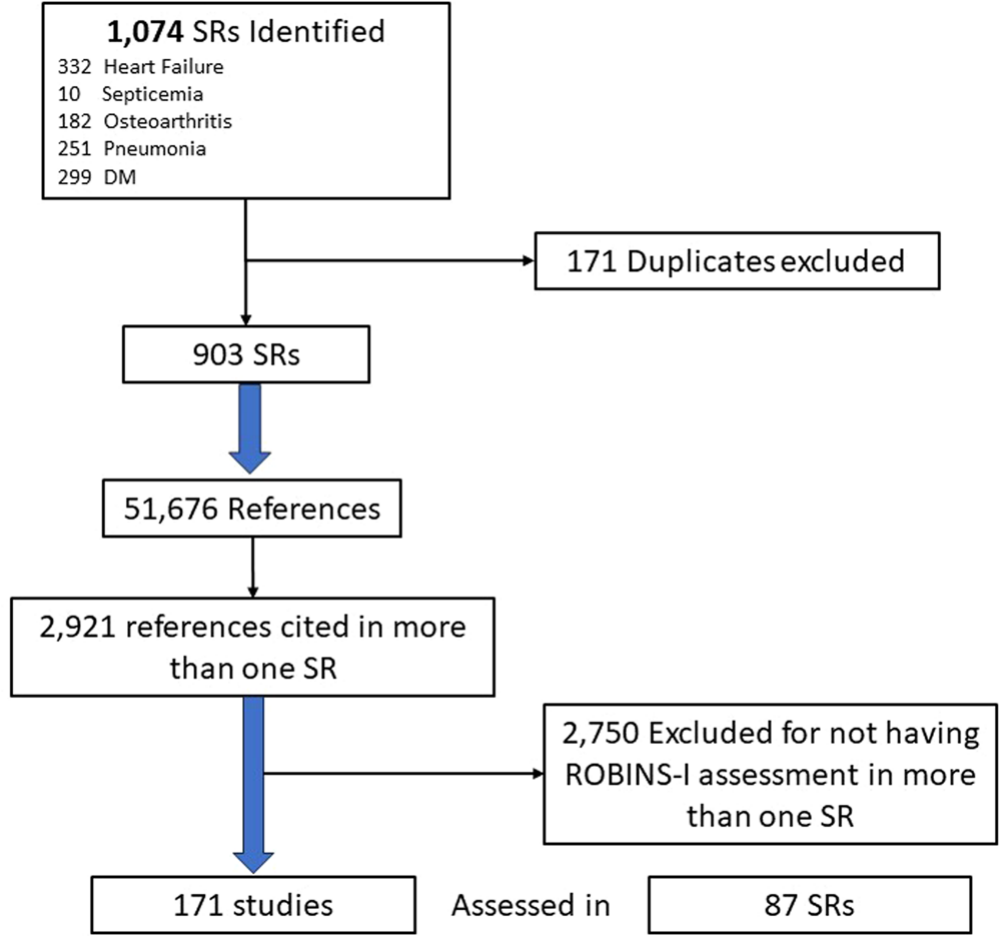
The process of study selection.

The search strategy is presented in the appendix and the study selection process is depicted in Figure 1. We identified 1074 systematic reviews citing the ROBINS‐I paper [5], and related to the top five inpatient diagnoses. Of these 903 SRs were eligible that collectively cited 51,676 references. Among these, 2921 references were cited in more than one SR. After excluding 2750 references that did not have ROBINS‐I assessments in multiple SRs, our final data set comprised 171 duplicated studies assessed using ROBINS‐I at least twice in 87 SRs. Among them, 12 studies have been assessed in three different SRs, while two studies were evaluated in four distinct SRs. The details of the included SRs and studies are provided in Supporting Information S1: Boxes 1 and 2 of the appendix, respectively.

### 3.1 Agreement in duplicated studies

The observed agreement on ROBINS‐I domains in duplicated studies ranged from 54.9% (missing data domain) to 70.3% (deviations from intended interventions domain), and was 63.0% for overall RoB assessment of the study. The kappa coefficient ranged from 0.131 (measurement of outcome domain) to 0.396 (domains of confounding and deviations from intended interventions), and was 0.404 for overall RoB assessment of the study. The Gwet coefficient ranged from 0.442 (missing data domain) to 0.645 (deviations from intended interventions domain), and was 0.533 for overall RoB assessment of the study. The results are presented in Table 2.

**Table 2:** Interconsensus agreement metrics in ROBINS‐I.

Domain	Observed agreement (%)	Kappa coefficient (95% confidence interval [CI])	Gwet coefficient (95% CI)
Confounding	60.5	0.396 (0.281, 0.511)	0.495 (0.398, 0.593)
Selection	58.6	0.285 (0.15, 0.42)	0.489 (0.392, 0.585)
Classification of interventions	60.5	0.274 (0.137, 0.41)	0.517 (0.421, 0.614)
Deviations from intended interventions	70.3	0.393 (0.263, 0.523)	0.645 (0.554, 0.736)
Missing data	54.9	0.222 (0.101, 0.342)	0.442 (0.341, 0.543)
Measurement of outcomes	55.6	0.131 (−0.08, 0.269)	0.464 (0.367, 0.561)
Reported result	64.8	0.326 (0.204, 0.449)	0.575 (0.483, 0.668)
Overall	63.0	0.404 (0.281, 0.526)	0.533 (0.427, 0.639)

## 4 DISCUSSION

RoB assessment is a key component of determining the trustworthiness of evidence and clinical decision‐making. The available tools for RoB assessment need to have good interrate reliability to ensure the accuracy of this important step, but assessing this reliability is often limited before RoB tools are released for wide use by systematic reviewers. We propose an approach for a post hoc assessment that is less time and resource intensive than other ways of tool validation. The approach depends on retrospectively evaluating RoB assessment of duplicated studies, which are studies that overlap systematic reviews.

Focusing on ROBINS‐I, the contemporary tool for evaluating RoB in nonrandomized studies, we found considerable variability in RoB assessment. This variability was observed in the different domains and in the overall global assessment at a study level. Since RoB reported in each published Cochrane systematic review was likely a result of consensus between two reviewers, the reported agreement should have stabilized and it should be higher than that for individual reviewers. Hence, the variability we observed across systematic reviews is more troubling.

Only a few studies have evaluated the reliability of ROBINS‐I. Jeyaraman et al. evaluated the interrater and interconsensus agreement of the tool and demonstrated that it varied across domains from poor to substantial [14]. The domain of bias due to missing data had the highest interrater agreement whereas selection bias and classification of intervention had the highest interconsensus agreement. The domain of confounding had the least agreement. Zhang et al. demonstrated that the interrater agreement on a single domain of ROBINS‐I varied and was highest for the two domains of bias in reporting the results and bias due to measurement of outcome, and was lowest for the domain of deviations from intended interventions [3]. Ingelstrom et al. have demonstrated that systematic reviews with lower credibility (assessed using AMSTAR 2, A MeaSurement Tool to Assess systematic Reviews) [15] have poorly applied ROBINS‐I [16]. Modifications and incorrect use of ROBINS‐I were common in their sample of reviews with low credibility.

### 4.1 Implications

The limited interrater reliability noted using ROBINS‐I is not surprising. The tool has been found to be complex with reported time for assessing a single study that can be as high as 7 h initially, reducing to 3 h after repeated use, and discussion time to reach consensus that can be as high as 40 min [3]. This highlights the need for tailoring the tool to the specific topic at hand and giving reviewers specific instructions on what content‐specific information to look for in the appraised study, and how to rate such specific information using the tool. Upfront piloting and training are crucial for reliable RoB assessment, which is suggested in ROBINS‐I manuals but perhaps is not explicitly or consistently done. In a pre/post study, it was demonstrated that agreement improved after guidance and training on the use of ROBINS‐I was provided, and so did evaluator burden [17].

In addition, special attention should be paid to the domains of missing data and outcome measurement, which had very low kappa scores. Systematic reviews should plan for how they will judge these two specific domains with a context‐specific perspective that their pairs of reviewers understand and consistently apply.

We also recommend the use of the proposed approach of post hoc evaluation of interrater reliability depending on duplicated studies because it reflects real‐world use of the tool, as opposed to prospective assessment in an artificial setting. This approach is also fast because it depends on existing RoB assessment, as opposed to conducting new RoB assessments to verify interrater reliability, which can be time and resource intensive. This approach can be applied to any RoB tool, such as those for randomized trials, diagnostic studies or noncomparative studies [18, 19, 20]. An obvious limitation for the proposed approach is that the tool must exist for a certain period of time to allow its use in systematic reviews and duplicated studies to exist.

## 5 CONCLUSION

A post hoc evaluation of RoB tools is feasible by focusing on duplicated studies that overlap in systematic reviews. The ROBINS‐I assessments demonstrated considerable variation in interconsensus reliability among various systematic reviews that assessed the same study, suggesting the need for more intensive upfront work to calibrate systematic reviewers about how to identify context‐specific information and agree on how to judge it.

## AUTHOR CONTRIBUTIONS

**Samer Saadi**: Conceptualization; data curation; formal analysis; investigation; methodology; project administration; supervision; validation; writing—original draft; writing—review and editing. **Bashar Hasan**: Conceptualization; data curation; formal analysis; investigation; methodology; project administration; validation; writing—original draft; writing—review and editing. **Adel Kanaan**: Data curation; formal analysis; investigation; writing—review and editing. **Mohamed Abusalih**: Data curation; formal analysis; investigation; writing—review and editing. **Zin Tarakji**: Data curation; formal analysis; investigation; writing—review and editing. **Mustafa Sadek**: Data curation; formal analysis; investigation; writing—review and editing. **Ayla Shamsi Basha**: Data curation; formal analysis; investigation; writing—review and editing. **Mohammed Firwana**: Data curation; formal analysis; investigation; writing—review and editing. **Zhen Wang**: Data curation; formal analysis; investigation; writing—review and editing. **M. Hassan Murad**: Conceptualization; data curation; formal analysis; investigation; methodology; project administration; supervision; validation; writing—original draft; writing—review and editing.

## CONFLICT OF INTEREST STATEMENT

The authors declare no conflict of interest.

## PEER REVIEW STATEMENT

The peer review history for this article is available at https://www.webofscience.com/api/gateway/wos/peer-review/10.1002/cesm.12094.

## Supporting information

Supporting information.

## Data Availability

Data are available in the article's Supporting Information.
