# Supplementary material for: Real‐world evaluation of interconsensus agreement of risk of bias tools: A case study using risk of bias in nonrandomized studies‐of interventions (ROBINS‐I)
Source: Cochrane Evid Synth Methods. 2024 Jun 26;2(7):e12094. doi: 10.1002/cesm.12094 (PMC11795881; doi:10.1002/cesm.12094)
Supplement: Supplementary file 1 — Supporting information. [file CESM-2-e12094-s001.docx]

**Search strategy:**

1. Heart Failure:

332 documents have cited:

Refined to: ( heart  AND failure )  AND  ( LIMIT-TO ( DOCTYPE ,  "re" ) )  AND  ( LIMIT-TO ( PUBSTAGE ,  "final" ) )  AND  ( LIMIT-TO ( SUBJAREA ,  "MEDI" ) )  AND  ( LIMIT-TO ( LANGUAGE ,  "English" ) )

[ROBINS-I: A tool for assessing risk of bias in non-randomised studies of interventions](https://www.scopus.com/record/display.uri?eid=2-s2.0-84991710934&origin=resultslist&sort=plf-f&cite=2-s2.0-84991710934&src=s&nlo=&nlr=&nls=&imp=t&sid=4158e3f52b764282665fef5f0dfed495&sot=cite&sdt=sisr&cluster=scosubtype%2c%22re%22%2ct%2bscopubstage%2c%22final%22%2ct%2bscosubjabbr%2c%22MEDI%22%2ct%2bscolang%2c%22English%22%2ct&sl=0&ref=%28heart+failure%29)

[Sterne J.A.](https://www.scopus.com/authid/detail.uri?origin=resultslist&authorId=56962728000&zone=), [Hernan M.A.](https://www.scopus.com/authid/detail.uri?origin=resultslist&authorId=7005914051&zone=), [Reeves B.C.](https://www.scopus.com/authid/detail.uri?origin=resultslist&authorId=7102248855&zone=), [Savovic J.](https://www.scopus.com/authid/detail.uri?origin=resultslist&authorId=57203657798&zone=), [Berkman N.D.](https://www.scopus.com/authid/detail.uri?origin=resultslist&authorId=7005212316&zone=), [Viswanathan M.](https://www.scopus.com/authid/detail.uri?origin=resultslist&authorId=7102067799&zone=), [Henry D.](https://www.scopus.com/authid/detail.uri?origin=resultslist&authorId=36012659800&zone=), (...), [Higgins J.P.](https://www.scopus.com/authid/detail.uri?origin=resultslist&authorId=57307017300&zone=)

(2016) BMJ (Online), 355 , art. no. i4919

1. Septicemia:

10 documents have cited:

Refined to: ( septicemia )  AND  ( LIMIT-TO ( PUBSTAGE ,  "final" ) )  AND  ( LIMIT-TO ( DOCTYPE ,  "re" ) )  AND  ( LIMIT-TO ( SUBJAREA ,  "MEDI" ) )  AND  ( LIMIT-TO ( LANGUAGE ,  "English" ) )

[ROBINS-I: A tool for assessing risk of bias in non-randomised studies of interventions](https://www.scopus.com/record/display.uri?eid=2-s2.0-84991710934&origin=resultslist&sort=plf-f&cite=2-s2.0-84991710934&src=s&nlo=&nlr=&nls=&imp=t&sid=7f13271f29ee06c0d5e60899c3e2b00e&sot=cite&sdt=cl&cluster=scopubstage%2c%22final%22%2ct%2bscosubtype%2c%22re%22%2ct%2bscosubjabbr%2c%22MEDI%22%2ct%2bscolang%2c%22English%22%2ct&sl=0&ref=%28septicemia%29)

[Sterne J.A.](https://www.scopus.com/authid/detail.uri?origin=resultslist&authorId=56962728000&zone=), [Hernan M.A.](https://www.scopus.com/authid/detail.uri?origin=resultslist&authorId=7005914051&zone=), [Reeves B.C.](https://www.scopus.com/authid/detail.uri?origin=resultslist&authorId=7102248855&zone=), [Savovic J.](https://www.scopus.com/authid/detail.uri?origin=resultslist&authorId=57203657798&zone=), [Berkman N.D.](https://www.scopus.com/authid/detail.uri?origin=resultslist&authorId=7005212316&zone=), [Viswanathan M.](https://www.scopus.com/authid/detail.uri?origin=resultslist&authorId=7102067799&zone=), [Henry D.](https://www.scopus.com/authid/detail.uri?origin=resultslist&authorId=36012659800&zone=), (...), [Higgins J.P.](https://www.scopus.com/authid/detail.uri?origin=resultslist&authorId=57307017300&zone=)

(2016) BMJ (Online), 355 , art. no. i4919

1. Osteoarthritis:

182 documents have cited:

Refined to: ( osteoarthritis )  AND  ( LIMIT-TO ( PUBSTAGE ,  "final" ) )  AND  ( LIMIT-TO ( DOCTYPE ,  "re" ) )  AND  ( LIMIT-TO ( SUBJAREA ,  "MEDI" ) )  AND  ( LIMIT-TO ( LANGUAGE ,  "English" ) )

[ROBINS-I: A tool for assessing risk of bias in non-randomised studies of interventions](https://www.scopus.com/record/display.uri?eid=2-s2.0-84991710934&origin=resultslist&sort=plf-f&cite=2-s2.0-84991710934&src=s&nlo=&nlr=&nls=&imp=t&sid=15f465972edaf426bb36d2e124f9e7f1&sot=cite&sdt=cl&cluster=scopubstage%2c%22final%22%2ct%2bscosubtype%2c%22re%22%2ct%2bscosubjabbr%2c%22MEDI%22%2ct%2bscolang%2c%22English%22%2ct&sl=0&ref=%28osteoarthritis%29)

[Sterne J.A.](https://www.scopus.com/authid/detail.uri?origin=resultslist&authorId=56962728000&zone=), [Hernan M.A.](https://www.scopus.com/authid/detail.uri?origin=resultslist&authorId=7005914051&zone=), [Reeves B.C.](https://www.scopus.com/authid/detail.uri?origin=resultslist&authorId=7102248855&zone=), [Savovic J.](https://www.scopus.com/authid/detail.uri?origin=resultslist&authorId=57203657798&zone=), [Berkman N.D.](https://www.scopus.com/authid/detail.uri?origin=resultslist&authorId=7005212316&zone=), [Viswanathan M.](https://www.scopus.com/authid/detail.uri?origin=resultslist&authorId=7102067799&zone=), [Henry D.](https://www.scopus.com/authid/detail.uri?origin=resultslist&authorId=36012659800&zone=), (...), [Higgins J.P.](https://www.scopus.com/authid/detail.uri?origin=resultslist&authorId=57307017300&zone=)

(2016) BMJ (Online), 355 , art. no. i4919

1. Pneumonia:

251 documents have cited:

Refined to: ( pneumonia )  AND  ( LIMIT-TO ( PUBSTAGE ,  "final" ) )  AND  ( LIMIT-TO ( DOCTYPE ,  "re" ) )  AND  ( LIMIT-TO ( SUBJAREA ,  "MEDI" ) )  AND  ( LIMIT-TO ( LANGUAGE ,  "English" ) )

[ROBINS-I: A tool for assessing risk of bias in non-randomised studies of interventions](https://www.scopus.com/record/display.uri?eid=2-s2.0-84991710934&origin=resultslist&sort=plf-f&cite=2-s2.0-84991710934&src=s&nlo=&nlr=&nls=&imp=t&sid=d168ab7c2bb2138a1b48d976b714ddd4&sot=cite&sdt=cl&cluster=scopubstage%2c%22final%22%2ct%2bscosubtype%2c%22re%22%2ct%2bscosubjabbr%2c%22MEDI%22%2ct%2bscolang%2c%22English%22%2ct&sl=0&ref=%28pneumonia%29)

[Sterne J.A.](https://www.scopus.com/authid/detail.uri?origin=resultslist&authorId=56962728000&zone=), [Hernan M.A.](https://www.scopus.com/authid/detail.uri?origin=resultslist&authorId=7005914051&zone=), [Reeves B.C.](https://www.scopus.com/authid/detail.uri?origin=resultslist&authorId=7102248855&zone=), [Savovic J.](https://www.scopus.com/authid/detail.uri?origin=resultslist&authorId=57203657798&zone=), [Berkman N.D.](https://www.scopus.com/authid/detail.uri?origin=resultslist&authorId=7005212316&zone=), [Viswanathan M.](https://www.scopus.com/authid/detail.uri?origin=resultslist&authorId=7102067799&zone=), [Henry D.](https://www.scopus.com/authid/detail.uri?origin=resultslist&authorId=36012659800&zone=), (...), [Higgins J.P.](https://www.scopus.com/authid/detail.uri?origin=resultslist&authorId=57307017300&zone=)

(2016) BMJ (Online), 355 , art. no. i4919

1. Diabetes Mellites:

299 documents have cited:

Refined to: ( diabetes  AND mellitus )  AND  ( LIMIT-TO ( PUBSTAGE ,  "final" ) )  AND  ( LIMIT-TO ( DOCTYPE ,  "re" ) )  AND  ( LIMIT-TO ( SUBJAREA ,  "MEDI" ) )  AND  ( LIMIT-TO ( LANGUAGE ,  "English" ) )

[ROBINS-I: A tool for assessing risk of bias in non-randomised studies of interventions](https://www.scopus.com/record/display.uri?eid=2-s2.0-84991710934&origin=resultslist&sort=plf-f&cite=2-s2.0-84991710934&src=s&nlo=&nlr=&nls=&imp=t&sid=83ff1629ecc8e75350f8d46aaed42ff4&sot=cite&sdt=cl&cluster=scopubstage%2c%22final%22%2ct%2bscosubtype%2c%22re%22%2ct%2bscosubjabbr%2c%22MEDI%22%2ct%2bscolang%2c%22English%22%2ct&sl=0&ref=%28diabetes+mellitus%29)

[Sterne J.A.](https://www.scopus.com/authid/detail.uri?origin=resultslist&authorId=56962728000&zone=), [Hernan M.A.](https://www.scopus.com/authid/detail.uri?origin=resultslist&authorId=7005914051&zone=), [Reeves B.C.](https://www.scopus.com/authid/detail.uri?origin=resultslist&authorId=7102248855&zone=), [Savovic J.](https://www.scopus.com/authid/detail.uri?origin=resultslist&authorId=57203657798&zone=), [Berkman N.D.](https://www.scopus.com/authid/detail.uri?origin=resultslist&authorId=7005212316&zone=), [Viswanathan M.](https://www.scopus.com/authid/detail.uri?origin=resultslist&authorId=7102067799&zone=), [Henry D.](https://www.scopus.com/authid/detail.uri?origin=resultslist&authorId=36012659800&zone=), (...), [Higgins J.P.](https://www.scopus.com/authid/detail.uri?origin=resultslist&authorId=57307017300&zone=)

(2016) BMJ (Online), 355 , art. no. i4919

**Box 1.** Included systematic reviews.

| 1. Eke AC, Sheffield J, Graham EM. 17alpha-Hydroxyprogesterone Caproate and the Risk of Glucose Intolerance in Pregnancy: A Systematic Review and Meta-analysis. *Obstet Gynecol* 2019;133(3):468-75. doi: 10.1097/AOG.0000000000003115 [published Online First: 2019/02/12]  2. Bellos I, Karageorgiou V, Pergialiotis V, et al. Acute kidney injury following the concurrent administration of antipseudomonal beta-lactams and vancomycin: a network meta-analysis. *Clin Microbiol Infect* 2020;26(6):696-705. doi: 10.1016/j.cmi.2020.03.019 [published Online First: 2020/03/31]  3. Martinez-Sobalvarro JV, Junior AAP, Pereira LB, et al. Antimicrobial stewardship for surgical antibiotic prophylaxis and surgical site infections: a systematic review. *Int J Clin Pharm* 2022;44(2):301-19. doi: 10.1007/s11096-021-01358-4 [published Online First: 2021/11/30]  4. Matsuzaki S, Ueda Y, Matsuzaki S, et al. Assisted Reproductive Technique and Abnormal Cord Insertion: A Systematic Review and Meta-Analysis. *Biomedicines* 2022;10(7) doi: 10.3390/biomedicines10071722 [published Online First: 2022/07/28]  5. Torabynasab K, Shahinfar H, Payandeh N, et al. Association between dietary caffeine, coffee, and tea consumption and depressive symptoms in adults: A systematic review and dose-response meta-analysis of observational studies. *Front Nutr* 2023;10:1051444. doi: 10.3389/fnut.2023.1051444 [published Online First: 2023/02/28]  6. Toews I, Lohner S, Kullenberg de Gaudry D, et al. Association between intake of non-sugar sweeteners and health outcomes: systematic review and meta-analyses of randomised and non-randomised controlled trials and observational studies. *BMJ* 2019;364:k4718. doi: 10.1136/bmj.k4718 [published Online First: 2019/01/04]  7. Matsuzaki S, Nagase Y, Ueda Y, et al. The association of endometriosis with placenta previa and postpartum hemorrhage: a systematic review and meta-analysis. *Am J Obstet Gynecol MFM* 2021;3(5):100417. doi: 10.1016/j.ajogmf.2021.100417 [published Online First: 2021/06/08]  8. Belk JW, Kraeutler MJ, Thon SG, et al. Augmentation of Meniscal Repair With Platelet-Rich Plasma: A Systematic Review of Comparative Studies. *Orthop J Sports Med* 2020;8(6):2325967120926145. doi: 10.1177/2325967120926145 [published Online First: 2020/07/01]  9. Liu X, Zhang Y, Lu L, et al. Benefits of high-dose intravenous immunoglobulin on mortality in patients with severe COVID-19: An updated systematic review and meta-analysis. *Front Immunol* 2023;14:1116738. doi: 10.3389/fimmu.2023.1116738 [published Online First: 2023/02/10]  10. Gottlieb M, Dyer S, Peksa GD. Beta-blockade for the treatment of cardiac arrest due to ventricular fibrillation or pulseless ventricular tachycardia: A systematic review and meta-analysis. *Resuscitation* 2020;146:118-25. doi: 10.1016/j.resuscitation.2019.11.019 [published Online First: 2019/12/04]  11. Gulea C, Zakeri R, Alderman V, et al. Beta-blocker therapy in patients with COPD: a systematic literature review and meta-analysis with multiple treatment comparison. *Respir Res* 2021;22(1):64. doi: 10.1186/s12931-021-01661-8 [published Online First: 2021/02/25]  12. Ruzieh M, Baugh AD, Al Jebbawi L, et al. Beta-blocker use in patients with chronic obstructive pulmonary disease: A systematic review: A systematic review of betaB in COPD. *Trends Cardiovasc Med* 2023;33(1):53-61. doi: 10.1016/j.tcm.2021.11.004 [published Online First: 2021/12/03]  13. Zaffagnini S, Poggi A, Reale D, et al. Biologic Augmentation Reduces the Failure Rate of Meniscal Repair: A Systematic Review and Meta-analysis. *Orthop J Sports Med* 2021;9(2):2325967120981627. doi: 10.1177/2325967120981627 [published Online First: 2021/03/13]  14. Zlatanovic P, Jovanovic A, Tripodi P, et al. Chimney Versus Fenestrated Endovascular Versus Open Repair for Juxta/Pararenal Abdominal Aortic Aneurysms: Systematic Review and Network Meta-analysis of the Short-term Results. *World J Surg* 2023;47(3):803-23. doi: 10.1007/s00268-022-06829-x [published Online First: 2022/11/24]  15. Zlatanovic P, Jovanovic A, Tripodi P, et al. Chimney vs. Fenestrated Endovascular vs. Open Repair for Juxta/Pararenal Abdominal Aortic Aneurysms: Systematic Review and Network Meta-Analysis of the Medium-Term Results. *J Clin Med* 2022;11(22) doi: 10.3390/jcm11226779 [published Online First: 2022/11/27]  16. Zhang J, Wang X, Liu X, et al. Comparative effectiveness and safety of direct acting oral anticoagulants in nonvalvular atrial fibrillation for stroke prevention: a systematic review and meta-analysis. *Eur J Epidemiol* 2021;36(8):793-812. doi: 10.1007/s10654-021-00751-7 [published Online First: 2021/05/17]  17. Kim MS, An MH, Kim WJ, et al. Comparative efficacy and safety of pharmacological interventions for the treatment of COVID-19: A systematic review and network meta-analysis. *PLoS Med* 2020;17(12):e1003501. doi: 10.1371/journal.pmed.1003501 [published Online First: 2020/12/31]  18. Kim JH, Ahn C, Park Y, et al. Comparison of out-of-hospital cardiac arrests during the COVID-19 pandemic with those before the pandemic: an updated systematic review and meta-analysis. *Front Public Health* 2023;11:1180511. doi: 10.3389/fpubh.2023.1180511 [published Online First: 2023/05/26]  19. Gad H, Al-Muhannadi H, Mussleman P, et al. Continuous subcutaneous insulin infusion versus multiple daily insulin injections in patients with Type 1 diabetes mellitus who fast during Ramadan: A systematic review and meta-analysis. *Diabetes Res Clin Pract* 2019;151:265-74. doi: 10.1016/j.diabres.2019.02.019 [published Online First: 2019/03/03]  20. Wang Y, Huo P, Dai R, et al. Convalescent plasma may be a possible treatment for COVID-19: A systematic review. *Int Immunopharmacol* 2021;91:107262. doi: 10.1016/j.intimp.2020.107262 [published Online First: 2020/12/19]  21. Piechotta V, Chai KL, Valk SJ, et al. Convalescent plasma or hyperimmune immunoglobulin for people with COVID-19: a living systematic review. *Cochrane Database Syst Rev* 2020;7(7):CD013600. doi: 10.1002/14651858.CD013600.pub2 [published Online First: 2020/07/11]  22. Aviani JK, Halim D, Soeroto AY, et al. Current views on the potentials of convalescent plasma therapy (CPT) as Coronavirus disease 2019 (COVID-19) treatment: A systematic review and meta-analysis based on recent studies and previous respiratory pandemics. *Rev Med Virol* 2021;31(6):e2225. doi: 10.1002/rmv.2225 [published Online First: 2021/02/24]  23. Pomozi E, Nagy R, Fehervari P, et al. Direct Oral Anticoagulants as the First Choice of Anticoagulation for Patients with Peripheral Artery Disease to Prevent Adverse Vascular Events: A Systematic Review and Meta-Analysis. *J Cardiovasc Dev Dis* 2023;10(2) doi: 10.3390/jcdd10020065 [published Online First: 2023/02/25]  24. Liang YS, Yu KC, Wong CS, et al. Does Surgery Reduce the Risk of Complications Among Patients with Multiple Rib Fractures? A Meta-analysis. *Clin Orthop Relat Res* 2019;477(1):193-205. doi: 10.1097/CORR.0000000000000495 [published Online First: 2018/09/25]  25. Fiolet T, Guihur A, Rebeaud ME, et al. Effect of hydroxychloroquine with or without azithromycin on the mortality of coronavirus disease 2019 (COVID-19) patients: a systematic review and meta-analysis. *Clin Microbiol Infect* 2021;27(1):19-27. doi: 10.1016/j.cmi.2020.08.022 [published Online First: 2020/08/30]  26. Matiashova L, Shanker A, Isayeva G. The effect of intermittent fasting on mortality in patients with type 2 diabetes and metabolic disease with high cardiovascular risk: a systematic review. *Clinical Diabetology* 2021;10(3):284-89. doi: 10.5603/DK.a2021.0016  27. Auener SL, Remers TEP, van Dulmen SA, et al. The Effect of Noninvasive Telemonitoring for Chronic Heart Failure on Health Care Utilization: Systematic Review. *J Med Internet Res* 2021;23(9):e26744. doi: 10.2196/26744 [published Online First: 2021/09/30]  28. Gad H, Al-Muhannadi H, Purra H, et al. The effect of Ramadan focused education on patients with type 2 diabetes: A systematic review and meta-analysis. *Diabetes Res Clin Pract* 2020;162:108122. doi: 10.1016/j.diabres.2020.108122 [published Online First: 2020/03/21]  29. Buckley BJR, Lane DA, Calvert P, et al. Effectiveness and Safety of Apixaban in over 3.9 Million People with Atrial Fibrillation: A Systematic Review and Meta-Analysis. *J Clin Med* 2022;11(13) doi: 10.3390/jcm11133788 [published Online First: 2022/07/10]  30. Coleman BL, Sanderson R, Haag MDM, et al. Effectiveness of the MF59-adjuvanted trivalent or quadrivalent seasonal influenza vaccine among adults 65 years of age or older, a systematic review and meta-analysis. *Influenza Other Respir Viruses* 2021;15(6):813-23. doi: 10.1111/irv.12871 [published Online First: 2021/06/04]  31. Scquizzato T, Landoni G, Paoli A, et al. Effects of COVID-19 pandemic on out-of-hospital cardiac arrests: A systematic review. *Resuscitation* 2020;157:241-47. doi: 10.1016/j.resuscitation.2020.10.020 [published Online First: 2020/11/02]  32. Hung DT, Ghula S, Aziz JMA, et al. The efficacy and adverse effects of favipiravir on patients with COVID-19: A systematic review and meta-analysis of published clinical trials and observational studies. *Int J Infect Dis* 2022;120:217-27. doi: 10.1016/j.ijid.2022.04.035 [published Online First: 2022/04/27]  33. Das RR, Jaiswal N, Dev N, et al. Efficacy and Safety of Anti-malarial Drugs (Chloroquine and Hydroxy-Chloroquine) in Treatment of COVID-19 Infection: A Systematic Review and Meta-Analysis. *Front Med (Lausanne)* 2020;7:482. doi: 10.3389/fmed.2020.00482 [published Online First: 2020/08/28]  34. Rhee TM, Lee SR, Choi EK, et al. Efficacy and Safety of Oral Anticoagulants for Atrial Fibrillation Patients With Chronic Kidney Disease: A Systematic Review and Meta-Analysis. *Front Cardiovasc Med* 2022;9:885548. doi: 10.3389/fcvm.2022.885548 [published Online First: 2022/06/28]  35. Focosi D, Franchini M, Tuccori M, et al. Efficacy of High-Dose Polyclonal Intravenous Immunoglobulin in COVID-19: A Systematic Review. *Vaccines (Basel)* 2022;10(1) doi: 10.3390/vaccines10010094 [published Online First: 2022/01/23]  36. Miraglia D, Miguel LA, Alonso W. Esmolol in the management of pre-hospital refractory ventricular fibrillation: A systematic review and meta-analysis. *Am J Emerg Med* 2020;38(9):1921-34. doi: 10.1016/j.ajem.2020.05.083 [published Online First: 2020/08/11]  37. Pizarro Ângela Maria Vilaça Pereira de A, Martins Maria Rosário O, Simões Jorge A. Expanding Primary Care to Pharmaceutical Patient Care in Diabetes Mellitus Type 2 through the European Union’s Community Pharmacies, between 2008 and 2018: A Systematic Review. *Portuguese Journal of Public Health* 2019;37(2-3):100-18. doi: 10.1159/000506261  38. Sadeq AA, Hasan SS, AbouKhater N, et al. Exploring Antimicrobial Stewardship Influential Interventions on Improving Antibiotic Utilization in Outpatient and Inpatient Settings: A Systematic Review and Meta-Analysis. *Antibiotics (Basel)* 2022;11(10) doi: 10.3390/antibiotics11101306 [published Online First: 2022/10/28]  39. Pizarro Ângela Maria Vilaça Pereira de A, Martins Maria Rosário O, Simões Jorge A. Exploring the Policies Applied to Pharmaceutical Care Practice for Type 2 Diabetes over the Last Decade in European Community Pharmacies. *Portuguese Journal of Public Health* 2021;39(2):103-18. doi: 10.1159/000519498  40. Holmberg MJ, Geri G, Wiberg S, et al. Extracorporeal cardiopulmonary resuscitation for cardiac arrest: A systematic review. *Resuscitation* 2018;131:91-100. doi: 10.1016/j.resuscitation.2018.07.029 [published Online First: 2018/08/01]  41. Ollivier B, Berger P, Depuydt C, et al. Good long-term survival and patient-reported outcomes after high tibial osteotomy for medial compartment osteoarthritis. *Knee Surg Sports Traumatol Arthrosc* 2021;29(11):3569-84. doi: 10.1007/s00167-020-06262-4 [published Online First: 2020/09/11]  42. Sebok J, Edel Z, Vancsa S, et al. Heat therapy shows benefit in patients with type 2 diabetes mellitus: a systematic review and meta-analysis. *Int J Hyperthermia* 2021;38(1):1650-59. doi: 10.1080/02656736.2021.2003445 [published Online First: 2021/11/23]  43. Hernandez AV, Roman YM, Pasupuleti V, et al. Hydroxychloroquine or Chloroquine for Treatment or Prophylaxis of COVID-19: A Living Systematic Review. *Ann Intern Med* 2020;173(4):287-96. doi: 10.7326/M20-2496 [published Online First: 2020/05/28]  44. Brigadoi G, Rossin S, Visentin D, et al. The impact of Antimicrobial Stewardship Programmes in paediatric emergency departments and primary care: a systematic review. *Ther Adv Infect Dis* 2023;10:20499361221141771. doi: 10.1177/20499361221141771 [published Online First: 2023/01/20]  45. Doyon-Plourde P, Fakih I, Tadount F, et al. Impact of influenza vaccination on healthcare utilization - A systematic review. *Vaccine* 2019;37(24):3179-89. doi: 10.1016/j.vaccine.2019.04.051 [published Online First: 2019/05/03]  46. Sarkar S, Khanna P, Singh AK. The Impact of Neutrophil-Lymphocyte Count Ratio in COVID-19: A Systematic Review and Meta-Analysis. *J Intensive Care Med* 2022;37(7):857-69. doi: 10.1177/08850666211045626 [published Online First: 2021/10/22]  47. Lee H, Ryu K, Sohn Y, et al. Impact on Patient Outcomes of Pharmacist Participation in Multidisciplinary Critical Care Teams: A Systematic Review and Meta-Analysis. *Crit Care Med* 2019;47(9):1243-50. doi: 10.1097/CCM.0000000000003830 [published Online First: 2019/05/29]  48. Leo DG, Buckley BJR, Chowdhury M, et al. Interactive Remote Patient Monitoring Devices for Managing Chronic Health Conditions: Systematic Review and Meta-analysis. *J Med Internet Res* 2022;24(11):e35508. doi: 10.2196/35508 [published Online First: 2022/11/04]  49. Cornelissen D, de Kunder S, Si L, et al. Interventions to improve adherence to anti-osteoporosis medications: an updated systematic review. *Osteoporos Int* 2020;31(9):1645-69. doi: 10.1007/s00198-020-05378-0 [published Online First: 2020/05/03]  50. Martin J, Viprey M, Castagne B, et al. Interventions to improve osteoporosis care: a systematic review and meta-analysis. *Osteoporos Int* 2020;31(3):429-46. doi: 10.1007/s00198-020-05308-0 [published Online First: 2020/01/30]  51. Marcec R, Dodig VM, Radanovic I, et al. Intravenous immunoglobulin (IVIg) therapy in hospitalised adult COVID-19 patients: A systematic review and meta-analysis. *Rev Med Virol* 2022;32(6):e2397. doi: 10.1002/rmv.2397 [published Online First: 2022/09/14]  52. Tadount F, Doyon-Plourde P, Rafferty E, et al. Is there a difference in the immune response, efficacy, effectiveness and safety of seasonal influenza vaccine in males and females? - A systematic review. *Vaccine* 2020;38(3):444-59. doi: 10.1016/j.vaccine.2019.10.091 [published Online First: 2019/11/13]  53. Blears EE, Morris J, Popp D, et al. Kidney Injury in Critically Ill Patients Treated with Vancomycin and Zosyn or an Alternative: A Systematic Review and Meta-Analysis. *Surg Infect (Larchmt)* 2022;23(6):516-24. doi: 10.1089/sur.2022.128 [published Online First: 2022/06/24]  54. Miraglia D, Miguel LA, Alonso W. Long-term neurologically intact survival after extracorporeal cardiopulmonary resuscitation for in-hospital or out-of-hospital cardiac arrest: A systematic review and meta-analysis. *Resusc Plus* 2020;4:100045. doi: 10.1016/j.resplu.2020.100045 [published Online First: 2021/07/06]  55. Batailler C, Fernandez A, Swan J, et al. MAKO CT-based robotic arm-assisted system is a reliable procedure for total knee arthroplasty: a systematic review. *Knee Surg Sports Traumatol Arthrosc* 2021;29(11):3585-98. doi: 10.1007/s00167-020-06283-z [published Online First: 2020/09/26]  56. Berton A, Gulotta LV, Longo UG, et al. Medialized versus Lateralized Center of Rotation in Reverse Total Shoulder Arthroplasty: A Systematic Review and Meta-Analysis. *J Clin Med* 2021;10(24) doi: 10.3390/jcm10245868 [published Online First: 2021/12/25]  57. Al Shamry A, Jegaden M, Ashafy S, et al. Minithoracotomy versus sternotomy in mitral valve surgery: meta-analysis from recent matched and randomized studies. *J Cardiothorac Surg* 2023;18(1):101. doi: 10.1186/s13019-023-02229-x [published Online First: 2023/04/07]  58. Sa M, Van den Eynde J, Cavalcanti LRP, et al. Mitral valve repair with minimally invasive approaches vs sternotomy: A meta-analysis of early and late results in randomized and matched observational studies. *J Card Surg* 2020;35(9):2307-23. doi: 10.1111/jocs.14799 [published Online First: 2020/07/16]  59. Trieu J, Gould DJ, Schilling C, et al. Patient-Reported Outcomes Following Total Knee Replacement in Patients <65 Years of Age-A Systematic Review and Meta-Analysis. *J Clin Med* 2020;9(10):1-17. doi: 10.3390/jcm9103150 [published Online First: 2020/10/03]  60. Woodland N, Takla A, Estee MM, et al. Patient-Reported Outcomes following Total Knee Replacement in Patients Aged 65 Years and Over-A Systematic Review. *J Clin Med* 2023;12(4) doi: 10.3390/jcm12041613 [published Online First: 2023/02/26]  61. Conforti A, Picarelli S, Carbone L, et al. Perinatal and obstetric outcomes in singleton pregnancies following fresh versus cryopreserved blastocyst transfer: a meta-analysis. *Reprod Biomed Online* 2021;42(2):401-12. doi: 10.1016/j.rbmo.2020.09.029 [published Online First: 2020/11/26]  62. Monmaturapoj T, Scott J, Smith P, et al. Pharmacist-led education-based antimicrobial stewardship interventions and their effect on antimicrobial use in hospital inpatients: a systematic review and narrative synthesis. *J Hosp Infect* 2021;115:93-116. doi: 10.1016/j.jhin.2021.06.003 [published Online First: 2021/06/19]  63. Matsuzaki S, Ueda Y, Nagase Y, et al. Placenta Accreta Spectrum Disorder Complicated with Endometriosis: Systematic Review and Meta-Analysis. *Biomedicines* 2022;10(2) doi: 10.3390/biomedicines10020390 [published Online First: 2022/02/26]  64. Pergialiotis V, Bellos I, Hatziagelaki E, et al. Progestogens for the prevention of preterm birth and risk of developing gestational diabetes mellitus: a meta-analysis. *Am J Obstet Gynecol* 2019;221(5):429-36 e5. doi: 10.1016/j.ajog.2019.05.033 [published Online First: 2019/05/28]  65. Nussbaumer-Streit B, Mayr V, Dobrescu AI, et al. Quarantine alone or in combination with other public health measures to control COVID-19: a rapid review. *Cochrane Database Syst Rev* 2020;4(4):CD013574. doi: 10.1002/14651858.CD013574 [published Online First: 2020/04/09]  66. Nussbaumer-Streit B, Mayr V, Dobrescu AI, et al. Quarantine alone or in combination with other public health measures to control COVID-19: a rapid review. *Cochrane Database Syst Rev* 2020;9(9):CD013574. doi: 10.1002/14651858.CD013574.pub2 [published Online First: 2021/05/08]  67. Cortegiani A, Ippolito M, Greco M, et al. Rationale and evidence on the use of tocilizumab in COVID-19: a systematic review. *Pulmonology* 2021;27(1):52-66. doi: 10.1016/j.pulmoe.2020.07.003 [published Online First: 2020/07/28]  68. Lee HW, Yoon CH, Jang EJ, et al. Renin-angiotensin system blocker and outcomes of COVID-19: a systematic review and meta-analysis. *Thorax* 2021;76(5):479-86. doi: 10.1136/thoraxjnl-2020-215322 [published Online First: 2021/01/29]  69. Kotecha P, Light A, Checcucci E, et al. Repurposing of drugs for COVID-19: a systematic review and meta-analysis. *Panminerva Med* 2022;64(1):96-114. doi: 10.23736/S0031-0808.20.04024-0 [published Online First: 2020/10/20]  70. Prada L, C DS, Baiao RA, et al. Risk of SARS-CoV-2 Infection and COVID-19 Severity Associated With Exposure to Nonsteroidal Anti-Inflammatory Drugs: Systematic Review and Meta-Analysis. *J Clin Pharmacol* 2021;61(12):1521-33. doi: 10.1002/jcph.1949 [published Online First: 2021/08/06]  71. Longo UG, Gulotta LV, De Salvatore S, et al. The Role of Humeral Neck-Shaft Angle in Reverse Total Shoulder Arthroplasty: 155 degrees versus <155 degrees -A Systematic Review. *J Clin Med* 2022;11(13) doi: 10.3390/jcm11133641 [published Online First: 2022/07/10]  72. Sarkar S, Kannan S, Khanna P, et al. Role of red blood cell distribution width, as a prognostic indicator in COVID-19: A systematic review and meta-analysis. *Rev Med Virol* 2022;32(2):e2264. doi: 10.1002/rmv.2264 [published Online First: 2021/06/07]  73. Bhowmick S, Dang A, Vallish BN, et al. Safety and Efficacy of Ivermectin and Doxycycline Monotherapy and in Combination in the Treatment of COVID-19: A Scoping Review. *Drug Saf* 2021;44(6):635-44. doi: 10.1007/s40264-021-01066-y [published Online First: 2021/04/18]  74. Castelli L, Galasso L, Mule A, et al. Sleep and spa therapies: What is the role of balneotherapy associated with exercise? A systematic review. *Front Physiol* 2022;13:964232. doi: 10.3389/fphys.2022.964232 [published Online First: 2022/08/30]  75. Sawyer E, Wullschleger M, Muller N, et al. Surgical Rib Fixation of Multiple Rib Fractures and Flail Chest: A Systematic Review and Meta-analysis. *J Surg Res* 2022;276:221-34. doi: 10.1016/j.jss.2022.02.055 [published Online First: 2022/04/08]  76. Giossi R, Menichelli D, Pani A, et al. A Systematic Review and a Meta-Analysis Comparing Prophylactic and Therapeutic Low Molecular Weight Heparins for Mortality Reduction in 32,688 COVID-19 Patients. *Front Pharmacol* 2021;12:698008. doi: 10.3389/fphar.2021.698008 [published Online First: 2021/09/21]  77. Luxton TN, King N, Walti C, et al. A Systematic Review of the Effect of Therapeutic Drug Monitoring on Patient Health Outcomes during Treatment with Carbapenems. *Antibiotics (Basel)* 2022;11(10) doi: 10.3390/antibiotics11101311 [published Online First: 2022/10/28]  78. Luxton T, King N, Walti C, et al. A systematic review of the effect of therapeutic drug monitoring on patient health outcomes during treatment with penicillins. *J Antimicrob Chemother* 2022;77(6):1532-41. doi: 10.1093/jac/dkac101 [published Online First: 2022/04/01]  79. Parker K, Hartemink J, Saha A, et al. A systematic review of the efficacy and safety of anticoagulants in advanced chronic kidney disease. *J Nephrol* 2022;35(8):2015-33. doi: 10.1007/s40620-022-01413-x [published Online First: 2022/08/26]  80. Comber L, E OM, Jordan K, et al. Systematic review of the efficacy, effectiveness and safety of high-dose seasonal influenza vaccines for the prevention of laboratory-confirmed influenza in individuals >/=18 years of age. *Rev Med Virol* 2023;33(3):e2330. doi: 10.1002/rmv.2330 [published Online First: 2022/02/05]  81. E OM, Comber L, Jordan K, et al. Systematic review of the efficacy, effectiveness and safety of MF59((R)) adjuvanted seasonal influenza vaccines for the prevention of laboratory-confirmed influenza in individuals >/=18 years of age. *Rev Med Virol* 2023;33(3):e2329. doi: 10.1002/rmv.2329 [published Online First: 2022/02/11]  82. Ping H, Wen J, Liu Y, et al. Unicompartmental knee arthroplasty is associated with lower pain levels but inferior range of motion, compared with high tibial osteotomy: a systematic overview of meta-analyses. *J Orthop Surg Res* 2022;17(1):425. doi: 10.1186/s13018-022-03319-7 [published Online First: 2022/09/25]  83. Zhang B, Qian H, Wu H, et al. Unicompartmental knee arthroplasty versus high tibial osteotomy for medial knee osteoarthritis: A systematic review and meta-analysis. *J Orthop Surg (Hong Kong)* 2023;31(1):10225536231162829. doi: 10.1177/10225536231162829 [published Online First: 2023/03/10]  84. Cao Z, Mai X, Wang J, et al. Unicompartmental Knee Arthroplasty vs High Tibial Osteotomy for Knee Osteoarthritis: A Systematic Review and Meta-Analysis. *J Arthroplasty* 2018;33(3):952-59. doi: 10.1016/j.arth.2017.10.025 [published Online First: 2017/12/06]  85. Cortegiani A, Ippolito M, Ingoglia G, et al. Update I. A systematic review on the efficacy and safety of chloroquine/hydroxychloroquine for COVID-19. *J Crit Care* 2020;59:176-90. doi: 10.1016/j.jcrc.2020.06.019 [published Online First: 2020/07/20]  86. Mazhar F, Hadi MA, Kow CS, et al. Use of hydroxychloroquine and chloroquine in COVID-19: How good is the quality of randomized controlled trials? *Int J Infect Dis* 2020;101:107-20. doi: 10.1016/j.ijid.2020.09.1470 [published Online First: 2020/10/03]  87. Bezerra Giordan L, Tong HL, Atherton JJ, et al. The Use of Mobile Apps for Heart Failure Self-management: Systematic Review of Experimental and Qualitative Studies. *JMIR Cardio* 2022;6(1):e33839. doi: 10.2196/33839 [published Online First: 2022/04/01] |
| --- |

**Box 2.** Included non-randomized studies.

| 1. Grossi EA, Goldman S, Wolfe JA, et al. Minithoracotomy for mitral valve repair improves inpatient and postdischarge economic savings. *J Thorac Cardiovasc Surg* 2014;148(6):2818-22 e1-3. doi: 10.1016/j.jtcvs.2014.08.029  10.1016/j.jtcvs.2014.08.029. Epub 2014 Aug 20. [published Online First: 2014/09/23]  2. Nishi H, Miyata H, Motomura N, et al. Propensity-matched analysis of minimally invasive mitral valve repair using a nationwide surgical database. *Surg Today* 2015;45(9):1144-52. doi: 10.1007/s00595-015-1210-7  10.1007/s00595-015-1210-7. Epub 2015 Jun 27. [published Online First: 2015/06/27]  3. Wang Q, Xi W, Gao Y, et al. Short-term outcomes of minimally invasive mitral valve repair: a propensity-matched comparison. *Interact Cardiovasc Thorac Surg* 2018;26(5):805-12. doi: 10.1093/icvts/ivx402  10.1093/icvts/ivx402. [published Online First: 2018/01/06]  4. Paparella D, Fattouch K, Moscarelli M, et al. Current trends in mitral valve surgery: A multicenter national comparison between full-sternotomy and minimally-invasive approach. *Int J Cardiol* 2020;306:147-51. doi: 10.1016/j.ijcard.2019.11.137  10.1016/j.ijcard.2019.11.137. Epub 2019 Nov 26. [published Online First: 2019/12/08]  5. Donas KP, Eisenack M, Panuccio G, et al. The role of open and endovascular treatment with fenestrated and chimney endografts for patients with juxtarenal aortic aneurysms. *J Vasc Surg* 2012;56(2):285-90. doi: 10.1016/j.jvs.2012.01.043  10.1016/j.jvs.2012.01.043. Epub 2012 May 2. [published Online First: 2012/05/05]  6. Lee JT, Lee GK, Chandra V, et al. Comparison of fenestrated endografts and the snorkel/chimney technique. *J Vasc Surg* 2014;60(4):849-56; discussion 56-7. doi: 10.1016/j.jvs.2014.03.255  10.1016/j.jvs.2014.03.255. Epub 2014 Apr 27. [published Online First: 2014/05/03]  7. Barilla D, Sobocinski J, Stilo F, et al. Juxtarenal aortic aneurysm with hostile neck anatomy: midterm results of minilaparotomy versus f-EVAR. *Int Angiol* 2014;33(5):466-73. [published Online First: 2014/10/09]  8. Shahverdyan R, Majd MP, Thul R, et al. F-EVAR does not Impair Renal Function more than Open Surgery for Juxtarenal Aortic Aneurysms: Single Centre Results. *Eur J Vasc Endovasc Surg* 2015;50(4):432-41. doi: 10.1016/j.ejvs.2015.04.028  10.1016/j.ejvs.2015.04.028. Epub 2015 Jun 19. [published Online First: 2015/06/24]  9. Maeda K, Ohki T, Kanaoka Y, et al. Comparison between Open and Endovascular Repair for the Treatment of Juxtarenal Abdominal Aortic Aneurysms: A Single-Center Experience with Midterm Results. *Ann Vasc Surg* 2017;41:96-104. doi: 10.1016/j.avsg.2016.08.045  10.1016/j.avsg.2016.08.045. Epub 2017 Feb 24. [published Online First: 2017/02/28]  10. Caradu C, Morin J, Poirier M, et al. Monocentric Evaluation of Chimney Versus Fenestrated Endovascular Aortic Repair for Juxtarenal Abdominal Aortic Aneurysm. *Ann Vasc Surg* 2017;40:28-38. doi: 10.1016/j.avsg.2016.09.013  10.1016/j.avsg.2016.09.013. Epub 2017 Feb 2. [published Online First: 2017/02/06]  11. Soler R, Bartoli MA, Faries C, et al. Fenestrated endovascular aneurysm repair and open surgical repair for the treatment of juxtarenal aortic aneurysms. *J Vasc Surg* 2019;70(3):683-90. doi: 10.1016/j.jvs.2018.11.041  10.1016/j.jvs.2018.11.041. Epub 2019 Mar 6. [published Online First: 2019/03/10]  12. O'Donnell TFX, Boitano LT, Deery SE, et al. Open Versus Fenestrated Endovascular Repair of Complex Abdominal Aortic Aneurysms. *Ann Surg* 2020;271(5):969-77. doi: 10.1097/SLA.0000000000003094  10.1097/SLA.0000000000003094. [published Online First: 2019/02/06]  13. Menegolo M, Xodo A, Penzo M, et al. Open repair versus EVAR with parallel grafts in patients with juxtarenal abdominal aortic aneurysm excluded from fenestrated endografting. *J Cardiovasc Surg (Torino)* 2021;62(5):483-95. doi: 10.23736/S0021-9509.21.11833-6  10.23736/S0021-9509.21.11833-6. Epub 2021 Jun 18. [published Online First: 2021/06/19]  14. Banno H, Cochennec F, Marzelle J, et al. Comparison of fenestrated endovascular aneurysm repair and chimney graft techniques for pararenal aortic aneurysm. *J Vasc Surg* 2014;60(1):31-9. doi: 10.1016/j.jvs.2014.01.036  10.1016/j.jvs.2014.01.036. Epub 2014 Feb 20. [published Online First: 2014/02/25]  15. Guo X, Park Y, Freedman ND, et al. Sweetened beverages, coffee, and tea and depression risk among older US adults. *Plos One* 2014;9(4):e94715. doi: 10.1371/journal.pone.0094715  10.1371/journal.pone.0094715. eCollection 2014. [published Online First: 2014/04/20]  16. Chan YH, Lee HF, Li PR, et al. Effectiveness, safety, and major adverse limb events in atrial fibrillation patients with concomitant diabetes mellitus treated with non-vitamin K antagonist oral anticoagulants. *Cardiovasc Diabetol* 2020;19(1):63. doi: 10.1186/s12933-020-01043-2  10.1186/s12933-020-01043-2. [published Online First: 2020/05/15]  17. Shao Z, Feng Y, Zhong L, et al. Clinical efficacy of intravenous immunoglobulin therapy in critical ill patients with COVID-19: a multicenter retrospective cohort study. *Clin Transl Immunology* 2020;9(10):e1192. doi: 10.1002/cti2.1192 [published Online First: 2020/10/22]  18. Esen F, Ozcan PE, Orhun G, et al. Effects of adjunct treatment with intravenous immunoglobulins on the course of severe COVID-19: results from a retrospective cohort study. *Curr Med Res Opin* 2021;37(4):543-48. doi: 10.1080/03007995.2020.1856058  10.1080/03007995.2020.1856058. Epub 2021 Feb 14. [published Online First: 2020/11/26]  19. Huang C, Fei L, Li W, et al. Efficacy evaluation of intravenous immunoglobulin in non-severe patients with COVID-19: A retrospective cohort study based on propensity score matching. *Int J Infect Dis* 2021;105:525-31. doi: 10.1016/j.ijid.2021.01.009  10.1016/j.ijid.2021.01.009. Epub 2021 Jan 9. [published Online First: 2021/01/13]  20. Cao W, Liu X, Hong K, et al. High-Dose Intravenous Immunoglobulin in Severe Coronavirus Disease 2019: A Multicenter Retrospective Study in China. *Front Immunol* 2021;12:627844. doi: 10.3389/fimmu.2021.627844 [published Online First: 2021/03/09]  21. Liu J, Chen Y, Li R, et al. Intravenous immunoglobulin treatment for patients with severe COVID-19: a retrospective multicentre study. *Clin Microbiol Infect* 2021;27(10):1488-93. doi: 10.1016/j.cmi.2021.05.012  10.1016/j.cmi.2021.05.012. Epub 2021 May 19. [published Online First: 2021/05/22]  22. Hou X, Tian L, Zhou L, et al. Intravenous immunoglobulin-based adjuvant therapy for severe COVID-19: a single-center retrospective cohort study. *Virol J* 2021;18(1):101. doi: 10.1186/s12985-021-01575-3  10.1186/s12985-021-01575-3. [published Online First: 2021/05/23]  23. Ali HS, Elshafei MS, Saad MO, et al. Clinical outcomes of intravenous immunoglobulin therapy in COVID-19 related acute respiratory distress syndrome: a retrospective cohort study. *BMC Pulm Med* 2021;21(1):354. doi: 10.1186/s12890-021-01717-x  10.1186/s12890-021-01717-x. [published Online First: 2021/11/09]  24. Farrokhpour M, Rezaie N, Moradi N, et al. Infliximab and Intravenous Gammaglobulin in Hospitalized Severe COVID-19 Patients in Intensive Care Unit. *Arch Iran Med* 2021;24(2):139-43. doi: 10.34172/aim.2021.22 [published Online First: 2021/02/28]  25. Baldi E, Sechi GM, Mare C, et al. COVID-19 kills at home: the close relationship between the epidemic and the increase of out-of-hospital cardiac arrests. *Eur Heart J* 2020;41(32):3045-54. doi: 10.1093/eurheartj/ehaa508  10.1093/eurheartj/ehaa508. [published Online First: 2020/06/21]  26. Cho JW, Jung H, Lee MJ, et al. Preparedness of personal protective equipment and implementation of new CPR strategies for patients with out-of-hospital cardiac arrest in the COVID-19 era. *Resusc Plus* 2020;3:100015. doi: 10.1016/j.resplu.2020.100015  10.1016/j.resplu.2020.100015. Epub 2020 Jun 25. [published Online First: 2021/05/26]  27. Lai PH, Lancet EA, Weiden MD, et al. Characteristics Associated With Out-of-Hospital Cardiac Arrests and Resuscitations During the Novel Coronavirus Disease 2019 Pandemic in New York City. *JAMA Cardiol* 2020;5(10):1154-63. doi: 10.1001/jamacardio.2020.2488 [published Online First: 2020/06/20]  28. Marijon E, Karam N, Jost D, et al. Out-of-hospital cardiac arrest during the COVID-19 pandemic in Paris, France: a population-based, observational study. *Lancet Public Health* 2020;5(8):e437-e43. doi: 10.1016/S2468-2667(20)30117-1 [published Online First: 2020/05/31]  29. Gottlieb SS, McCarter RJ, Vogel RA. Effect of beta-blockade on mortality among high-risk and low-risk patients after myocardial infarction. *N Engl J Med* 1998;339(8):489-97. doi: 10.1056/NEJM199808203390801  10.1056/NEJM199808203390801. [published Online First: 1998/08/26]  30. Sin DD, McAlister FA. The effects of beta-blockers on morbidity and mortality in a population-based cohort of 11,942 elderly patients with heart failure. *Am J Med* 2002;113(8):650-6. doi: 10.1016/s0002-9343(02)01346-3  10.1016/s0002-9343(02)01346-3. [published Online First: 2002/12/31]  31. van Gestel YR, Hoeks SE, Sin DD, et al. Impact of cardioselective beta-blockers on mortality in patients with chronic obstructive pulmonary disease and atherosclerosis. *Am J Respir Crit Care Med* 2008;178(7):695-700. doi: 10.1164/rccm.200803-384OC  10.1164/rccm.200803-384OC. Epub 2008 Jun 19. [published Online First: 2008/06/21]  32. Rutten FH, Zuithoff NP, Hak E, et al. Beta-blockers may reduce mortality and risk of exacerbations in patients with chronic obstructive pulmonary disease. *Arch Intern Med* 2010;170(10):880-7. doi: 10.1001/archinternmed.2010.112  10.1001/archinternmed.2010.112. [published Online First: 2010/05/26]  33. Short PM, Lipworth SI, Elder DH, et al. Effect of beta blockers in treatment of chronic obstructive pulmonary disease: a retrospective cohort study. *BMJ* 2011;342(7806):d2549. doi: 10.1136/bmj.d2549  10.1136/bmj.d2549. [published Online First: 2011/05/12]  34. Ekstrom MP, Hermansson AB, Strom KE. Effects of cardiovascular drugs on mortality in severe chronic obstructive pulmonary disease. *Am J Respir Crit Care Med* 2013;187(7):715-20. doi: 10.1164/rccm.201208-1565OC  10.1164/rccm.201208-1565OC. [published Online First: 2013/01/19]  35. Farland MZ, Peters CJ, Williams JD, et al. beta-Blocker use and incidence of chronic obstructive pulmonary disease exacerbations. *Ann Pharmacother* 2013;47(5):651-6. doi: 10.1345/aph.1R600  10.1345/aph.1R600. Epub 2013 Apr 12. [published Online First: 2013/04/16]  36. Quint JK, Herrett E, Bhaskaran K, et al. Effect of beta blockers on mortality after myocardial infarction in adults with COPD: population based cohort study of UK electronic healthcare records. *BMJ* 2013;347:f6650. doi: 10.1136/bmj.f6650 [published Online First: 2013/11/26]  37. Bhatt SP, Wells JM, Kinney GL, et al. beta-Blockers are associated with a reduction in COPD exacerbations. *Thorax* 2016;71(1):8-14. doi: 10.1136/thoraxjnl-2015-207251  10.1136/thoraxjnl-2015-207251. Epub 2015 Aug 17. [published Online First: 2015/08/19]  38. Su TH, Chang SH, Kuo CF, et al. beta-blockers after acute myocardial infarction in patients with chronic obstructive pulmonary disease: A nationwide population-based observational study. *Plos One* 2019;14(3):e0213187. doi: 10.1371/journal.pone.0213187  10.1371/journal.pone.0213187. eCollection 2019. [published Online First: 2019/03/06]  39. Maltais F, Buhl R, Koch A, et al. beta-Blockers in COPD: A Cohort Study From the TONADO Research Program. *Chest* 2018;153(6):1315-25. doi: 10.1016/j.chest.2018.01.008  10.1016/j.chest.2018.01.008. Epub 2018 Jan 31. [published Online First: 2018/01/23]  40. Hawkins NM, Huang Z, Pieper KS, et al. Chronic obstructive pulmonary disease is an independent predictor of death but not atherosclerotic events in patients with myocardial infarction: analysis of the Valsartan in Acute Myocardial Infarction Trial (VALIANT). *Eur J Heart Fail* 2009;11(3):292-8. doi: 10.1093/eurjhf/hfp001  10.1093/eurjhf/hfp001. Epub 2009 Jan 27. [published Online First: 2009/01/30]  41. Amir O, Ben-Gal T, Weinstein JM, et al. Evaluation of remote dielectric sensing (ReDS) technology-guided therapy for decreasing heart failure re-hospitalizations. *Int J Cardiol* 2017;240:279-84. doi: 10.1016/j.ijcard.2017.02.120  10.1016/j.ijcard.2017.02.120. Epub 2017 Mar 3. [published Online First: 2017/03/28]  42. Coleman CI, Kreutz R, Sood NA, et al. Rivaroxaban Versus Warfarin in Patients With Nonvalvular Atrial Fibrillation and Severe Kidney Disease or Undergoing Hemodialysis. *Am J Med* 2019;132(9):1078-83. doi: 10.1016/j.amjmed.2019.04.013  10.1016/j.amjmed.2019.04.013. Epub 2019 May 2. [published Online First: 2019/05/06]  43. Weir MR, Ashton V, Moore KT, et al. Rivaroxaban versus warfarin in patients with nonvalvular atrial fibrillation and stage IV-V chronic kidney disease. *Am Heart J* 2020;223:3-11. doi: 10.1016/j.ahj.2020.01.010  10.1016/j.ahj.2020.01.010. Epub 2020 Jan 22. [published Online First: 2020/03/01]  44. Wetmore JB, Roetker NS, Yan H, et al. Direct-Acting Oral Anticoagulants Versus Warfarin in Medicare Patients With Chronic Kidney Disease and Atrial Fibrillation. *Stroke* 2020;51(8):2364-73. doi: 10.1161/STROKEAHA.120.028934  10.1161/STROKEAHA.120.028934. Epub 2020 Jul 9. [published Online First: 2020/07/10]  45. Laugesen EK, Staerk L, Carlson N, et al. Non-vitamin K antagonist oral anticoagulants vs. vitamin-K antagonists in patients with atrial fibrillation and chronic kidney disease: a nationwide cohort study. *Thromb J* 2019;17(1):21. doi: 10.1186/s12959-019-0211-y  10.1186/s12959-019-0211-y. eCollection 2019. [published Online First: 2019/11/19]  46. Shin JI, Secora A, Alexander GC, et al. Risks and Benefits of Direct Oral Anticoagulants across the Spectrum of GFR among Incident and Prevalent Patients with Atrial Fibrillation. *Clin J Am Soc Nephrol* 2018;13(8):1144-52. doi: 10.2215/CJN.13811217  10.2215/CJN.13811217. Epub 2018 Jul 12. [published Online First: 2018/07/14]  47. Chang SH, Wu CV, Yeh YH, et al. Efficacy and Safety of Oral Anticoagulants in Patients With Atrial Fibrillation and Stages 4 or 5 Chronic Kidney Disease. *Am J Med* 2019;132(11):1335-43 e6. doi: 10.1016/j.amjmed.2019.06.006  10.1016/j.amjmed.2019.06.006. Epub 2019 Jul 3. [published Online First: 2019/07/07]  48. Buckley MS, Hartsock NC, Berry AJ, et al. Comparison of acute kidney injury risk associated with vancomycin and concomitant piperacillin/tazobactam or cefepime in the intensive care unit. *J Crit Care* 2018;48:32-38. doi: 10.1016/j.jcrc.2018.08.007  10.1016/j.jcrc.2018.08.007. Epub 2018 Aug 11. [published Online First: 2018/09/03]  49. Hammond DA, Smith MN, Painter JT, et al. Comparative Incidence of Acute Kidney Injury in Critically Ill Patients Receiving Vancomycin with Concomitant Piperacillin-Tazobactam or Cefepime: A Retrospective Cohort Study. *Pharmacotherapy* 2016;36(5):463-71. doi: 10.1002/phar.1738 [published Online First: 2016/03/10]  50. Jeon N, Staley B, Klinker KP, et al. Acute kidney injury risk associated with piperacillin/tazobactam compared with cefepime during vancomycin therapy in hospitalised patients: a cohort study stratified by baseline kidney function. *Int J Antimicrob Agents* 2017;50(1):63-67. doi: 10.1016/j.ijantimicag.2017.02.023 [published Online First: 2017/05/20]  51. Al Yami MS. Comparison of the incidence of acute kidney injury during treatment with vancomycin in combination with piperacillin-tazobactam or with meropenem. *J Infect Public Health* 2017;10(6):770-73. doi: 10.1016/j.jiph.2016.11.007 [published Online First: 2017/02/18]  52. Hundeshagen G, Herndon DN, Capek KD, et al. Co-administration of vancomycin and piperacillin-tazobactam is associated with increased renal dysfunction in adult and pediatric burn patients. *Crit Care* 2017;21(1):318. doi: 10.1186/s13054-017-1899-3 [published Online First: 2017/12/22]  53. Rutter WC, Burgess DS. Acute Kidney Injury in Patients Treated with IV Beta-Lactam/Beta-Lactamase Inhibitor Combinations. *Pharmacotherapy* 2017;37(5):593-98. doi: 10.1002/phar.1918 [published Online First: 2017/03/02]  54. Vinogradova Y, Coupland C, Hill T, et al. Risks and benefits of direct oral anticoagulants versus warfarin in a real world setting: cohort study in primary care. *BMJ* 2018;362:k2505. doi: 10.1136/bmj.k2505  10.1136/bmj.k2505. [published Online First: 2018/07/06]  55. Lee SR, Choi EK, Kwon S, et al. Effectiveness and Safety of Contemporary Oral Anticoagulants Among Asians With Nonvalvular Atrial Fibrillation. *Stroke* 2019;50(8):2245-49. doi: 10.1161/STROKEAHA.119.025536  10.1161/STROKEAHA.119.025536. Epub 2019 Jun 18. [published Online First: 2019/06/19]  56. Lip GYH, Keshishian A, Li X, et al. Effectiveness and Safety of Oral Anticoagulants Among Nonvalvular Atrial Fibrillation Patients. *Stroke* 2018;49(12):2933-44. doi: 10.1161/STROKEAHA.118.020232  10.1161/STROKEAHA.118.020232. [published Online First: 2018/12/21]  57. Jansson M, Sjalander S, Sjogren V, et al. Direct comparisons of effectiveness and safety of treatment with Apixaban, Dabigatran and Rivaroxaban in atrial fibrillation. *Thromb Res* 2020;185:135-41. doi: 10.1016/j.thromres.2019.11.010  10.1016/j.thromres.2019.11.010. Epub 2019 Nov 13. [published Online First: 2019/12/10]  58. Rutherford OW, Jonasson C, Ghanima W, et al. Comparison of dabigatran, rivaroxaban, and apixaban for effectiveness and safety in atrial fibrillation: a nationwide cohort study. *Eur Heart J Cardiovasc Pharmacother* 2020;6(2):75-85. doi: 10.1093/ehjcvp/pvz086  10.1093/ehjcvp/pvz086. [published Online First: 2020/01/17]  59. Mueller T, Alvarez-Madrazo S, Robertson C, et al. Comparative safety and effectiveness of direct oral anticoagulants in patients with atrial fibrillation in clinical practice in Scotland. *Br J Clin Pharmacol* 2019;85(2):422-31. doi: 10.1111/bcp.13814  10.1111/bcp.13814. Epub 2018 Dec 18. [published Online First: 2018/11/14]  60. Adeboyeje G, Sylwestrzak G, Barron JJ, et al. Major bleeding risk during anticoagulation with warfarin, dabigatran, apixaban, or rivaroxaban in patients with nonvalvular atrial fibrillation. *Journal of Managed Care and Specialty Pharmacy* 2017;23(9):968-78. doi: 10.18553/jmcp.2017.23.9.968  61. Tepper PG, Mardekian J, Masseria C, et al. Real-world comparison of bleeding risks among non-valvular atrial fibrillation patients prescribed apixaban, dabigatran, or rivaroxaban. *Plos One* 2018;13(11):e0205989. doi: 10.1371/journal.pone.0205989  10.1371/journal.pone.0205989. eCollection 2018. [published Online First: 2018/11/02]  62. Villines TC, Ahmad A, Petrini M, et al. Comparative safety and effectiveness of dabigatran vs. rivaroxaban and apixaban in patients with non-valvular atrial fibrillation: a retrospective study from a large healthcare system. *Eur Heart J Cardiovasc Pharmacother* 2019;5(2):80-90. doi: 10.1093/ehjcvp/pvy044  10.1093/ehjcvp/pvy044. [published Online First: 2018/12/01]  63. Amin A, Keshishian A, Vo L, et al. Real-world comparison of all-cause hospitalizations, hospitalizations due to stroke and major bleeding, and costs for non-valvular atrial fibrillation patients prescribed oral anticoagulants in a US health plan. *Journal of Medical Economics* 2018;21(3):244-53. doi: 10.1080/13696998.2017.1394866  64. Amin A, Garcia Reeves AB, Li X, et al. Effectiveness and safety of oral anticoagulants in older adults with non-valvular atrial fibrillation and heart failure. *Plos One* 2019;14(3) doi: 10.1371/journal.pone.0213614  65. Andersson NW, Svanström H, Lund M, et al. Comparative effectiveness and safety of apixaban, dabigatran, and rivaroxaban in patients with non-valvular atrial fibrillation. *International Journal of Cardiology* 2018;268:113-19. doi: 10.1016/j.ijcard.2018.03.047  66. Deitelzweig S, Bruno A, Trocio J, et al. An early evaluation of bleeding-related hospital readmissions among hospitalized patients with nonvalvular atrial fibrillation treated with direct oral anticoagulants. *Curr Med Res Opin* 2016;32(3):573-82. doi: 10.1185/03007995.2015.1131676  10.1185/03007995.2015.1131676. Epub 2016 Jan 1. [published Online First: 2015/12/15]  67. Deitelzweig S, Luo X, Gupta K, et al. Comparison of effectiveness and safety of treatment with apixaban vs. other oral anticoagulants among elderly nonvalvular atrial fibrillation patients. *Curr Med Res Opin* 2017;33(10):1745-54. doi: 10.1080/03007995.2017.1334638  10.1080/03007995.2017.1334638. Epub 2017 Aug 29. [published Online First: 2017/08/30]  68. Hernandez I, Zhang Y, Saba S. Comparison of the Effectiveness and Safety of Apixaban, Dabigatran, Rivaroxaban, and Warfarin in Newly Diagnosed Atrial Fibrillation. *Am J Cardiol* 2017;120(10):1813-19. doi: 10.1016/j.amjcard.2017.07.092  10.1016/j.amjcard.2017.07.092. Epub 2017 Aug 8. [published Online First: 2017/09/03]  69. Lip GY, Keshishian A, Kamble S, et al. Real-world comparison of major bleeding risk among non-valvular atrial fibrillation patients initiated on apixaban, dabigatran, rivaroxaban, or warfarin. A propensity score matched analysis. *Thromb Haemost* 2016;116(5):975-86. doi: 10.1160/TH16-05-0403  10.1160/TH16-05-0403. Epub 2016 Aug 19. [published Online First: 2016/10/30]  70. Noseworthy PA, Yao X, Abraham NS, et al. Direct Comparison of Dabigatran, Rivaroxaban, and Apixaban for Effectiveness and Safety in Nonvalvular Atrial Fibrillation. *Chest* 2016;150(6):1302-12. doi: 10.1016/j.chest.2016.07.013  10.1016/j.chest.2016.07.013. Epub 2016 Sep 28. [published Online First: 2016/12/13]  71. Gong J, Ou J, Qiu X, et al. A Tool for Early Prediction of Severe Coronavirus Disease 2019 (COVID-19): A Multicenter Study Using the Risk Nomogram in Wuhan and Guangdong, China. *Clin Infect Dis* 2020;71(15):833-40. doi: 10.1093/cid/ciaa443 [published Online First: 2020/04/17]  72. Wang C, Deng R, Gou L, et al. Preliminary study to identify severe from moderate cases of COVID-19 using combined hematology parameters. *Ann Transl Med* 2020;8(9):593. doi: 10.21037/atm-20-3391  10.21037/atm-20-3391. [published Online First: 2020/06/23]  73. Park C, Otobo E, Ullman J, et al. Impact on Readmission Reduction Among Heart Failure Patients Using Digital Health Monitoring: Feasibility and Adoptability Study. *JMIR Med Inform* 2019;7(4):e13353. doi: 10.2196/13353  10.2196/13353. [published Online First: 2019/11/16]  74. Twigg MJ, Desborough JA, Bhattacharya D, et al. An audit of prescribing for type 2 diabetes in primary care: optimising the role of the community pharmacist in the primary healthcare team. *Prim Health Care Res Dev* 2013;14(3):315-9. doi: 10.1017/S1463423612000345  10.1017/S1463423612000345. Epub 2012 Jul 13. [published Online First: 2012/11/24]  75. Lemmens-Gruber R, Hahnenkamp C, Gossmann U, et al. Evaluation of educational needs in patients with diabetes mellitus in respect of medication use in Austria. *Int J Clin Pharm* 2012;34(3):490-500. doi: 10.1007/s11096-012-9636-0  10.1007/s11096-012-9636-0. Epub 2012 Apr 24. [published Online First: 2012/04/25]  76. Taylor J, Krska J, Mackridge A. A community pharmacy-based cardiovascular screening service: views of service users and the public. *Int J Pharm Pract* 2012;20(5):277-84. doi: 10.1111/j.2042-7174.2012.00190.x  10.1111/j.2042-7174.2012.00190.x. Epub 2012 Mar 9. [published Online First: 2012/09/08]  77. van Geffen EC, Philbert D, van Boheemen C, et al. Patients' satisfaction with information and experiences with counseling on cardiovascular medication received at the pharmacy. *Patient Educ Couns* 2011;83(3):303-9. doi: 10.1016/j.pec.2011.04.004  10.1016/j.pec.2011.04.004. Epub 2011 May 6. [published Online First: 2011/05/10]  78. Wirth F, Tabone F, Azzopardi LM, et al. Consumer perception of the community pharmacist and community pharmacy services in Malta. *Journal of Pharmaceutical Health Services Research* 2010;1(4):189-94. doi: 10.1111/j.1759-8893.2010.00034.x  79. Ayerbe L, Risco C, Ayis S. The association between treatment with heparin and survival in patients with Covid-19. *J Thromb Thrombolysis* 2020;50(2):298-301. doi: 10.1007/s11239-020-02162-z [published Online First: 2020/06/02]  80. Mancia G, Rea F, Ludergnani M, et al. Renin-Angiotensin-Aldosterone System Blockers and the Risk of Covid-19. *N Engl J Med* 2020;382(25):2431-40. doi: 10.1056/NEJMoa2006923  10.1056/NEJMoa2006923. Epub 2020 May 1. [published Online First: 2020/05/02]  81. Somers EC, Eschenauer GA, Troost JP, et al. Tocilizumab for treatment of mechanically ventilated patients with COVID-19. *medRxiv* 2020 doi: 10.1101/2020.05.29.20117358  10.1101/2020.05.29.20117358. Preprint. [published Online First: 2020/06/25]  82. Rosenberg ES, Dufort EM, Udo T, et al. Association of Treatment With Hydroxychloroquine or Azithromycin With In-Hospital Mortality in Patients With COVID-19 in New York State. *JAMA* 2020;323(24):2493-502. doi: 10.1001/jama.2020.8630  10.1001/jama.2020.8630. [published Online First: 2020/05/12]  83. Mercuro NJ, Yen CF, Shim DJ, et al. Risk of QT Interval Prolongation Associated With Use of Hydroxychloroquine With or Without Concomitant Azithromycin Among Hospitalized Patients Testing Positive for Coronavirus Disease 2019 (COVID-19). *JAMA Cardiol* 2020;5(9):1036-41. doi: 10.1001/jamacardio.2020.1834  10.1001/jamacardio.2020.1834. [published Online First: 2020/09/17]  84. Saleh M, Gabriels J, Chang D, et al. Effect of Chloroquine, Hydroxychloroquine, and Azithromycin on the Corrected QT Interval in Patients With SARS-CoV-2 Infection. *Circ Arrhythm Electrophysiol* 2020;13(6):e008662. doi: 10.1161/CIRCEP.120.008662 [published Online First: 2020/04/30]  85. Mahevas M, Tran VT, Roumier M, et al. Clinical efficacy of hydroxychloroquine in patients with covid-19 pneumonia who require oxygen: observational comparative study using routine care data. *BMJ* 2020;369:m1844. doi: 10.1136/bmj.m1844 [published Online First: 2020/05/16]  86. Shin TG, Choi JH, Jo IJ, et al. Extracorporeal cardiopulmonary resuscitation in patients with inhospital cardiac arrest: A comparison with conventional cardiopulmonary resuscitation. *Crit Care Med* 2011;39(1):1-7. doi: 10.1097/CCM.0b013e3181feb339  10.1097/CCM.0b013e3181feb339. [published Online First: 2010/11/09]  87. Kim SJ, Jung JS, Park JH, et al. An optimal transition time to extracorporeal cardiopulmonary resuscitation for predicting good neurological outcome in patients with out-of-hospital cardiac arrest: a propensity-matched study. *Crit Care* 2014;18(5):535. doi: 10.1186/s13054-014-0535-8  10.1186/s13054-014-0535-8. [published Online First: 2014/09/27]  88. Maekawa K, Tanno K, Hase M, et al. Extracorporeal cardiopulmonary resuscitation for patients with out-of-hospital cardiac arrest of cardiac origin: a propensity-matched study and predictor analysis. *Crit Care Med* 2013;41(5):1186-96. doi: 10.1097/CCM.0b013e31827ca4c8  10.1097/CCM.0b013e31827ca4c8. [published Online First: 2013/02/08]  89. Sakamoto T, Morimura N, Nagao K, et al. Extracorporeal cardiopulmonary resuscitation versus conventional cardiopulmonary resuscitation in adults with out-of-hospital cardiac arrest: a prospective observational study. *Resuscitation* 2014;85(6):762-8. doi: 10.1016/j.resuscitation.2014.01.031  10.1016/j.resuscitation.2014.01.031. Epub 2014 Feb 12. [published Online First: 2014/02/18]  90. Shin TG, Jo IJ, Sim MS, et al. Two-year survival and neurological outcome of in-hospital cardiac arrest patients rescued by extracorporeal cardiopulmonary resuscitation. *Int J Cardiol* 2013;168(4):3424-30. doi: 10.1016/j.ijcard.2013.04.183  10.1016/j.ijcard.2013.04.183. Epub 2013 May 9. [published Online First: 2013/05/15]  91. Siao FY, Chiu CC, Chiu CW, et al. Managing cardiac arrest with refractory ventricular fibrillation in the emergency department: Conventional cardiopulmonary resuscitation versus extracorporeal cardiopulmonary resuscitation. *Resuscitation* 2015;92:70-6. doi: 10.1016/j.resuscitation.2015.04.016  10.1016/j.resuscitation.2015.04.016. Epub 2015 Apr 29. [published Online First: 2015/05/06]  92. Driver BE, Debaty G, Plummer DW, et al. Use of esmolol after failure of standard cardiopulmonary resuscitation to treat patients with refractory ventricular fibrillation. *Resuscitation* 2014;85(10):1337-41. doi: 10.1016/j.resuscitation.2014.06.032  10.1016/j.resuscitation.2014.06.032. Epub 2014 Jul 14. [published Online First: 2014/07/19]  93. Zeng QL, Yu ZJ, Gou JJ, et al. Effect of Convalescent Plasma Therapy on Viral Shedding and Survival in Patients With Coronavirus Disease 2019. *J Infect Dis* 2020;222(1):38-43. doi: 10.1093/infdis/jiaa228  10.1093/infdis/jiaa228. [published Online First: 2020/04/30]  94. Beaton DE, Mamdani M, Zheng H, et al. Improvements in osteoporosis testing and care are found following the wide scale implementation of the Ontario Fracture Clinic Screening Program: An interrupted time series analysis. *Medicine (Baltimore)* 2017;96(48):e9012. doi: 10.1097/MD.0000000000009012  10.1097/MD.0000000000009012. [published Online First: 2018/01/10]  95. Seuffert P, Sagebien CA, McDonnell M, et al. Evaluation of osteoporosis risk and initiation of a nurse practitioner intervention program in an orthopedic practice. *Arch Osteoporos* 2016;11(1):10. doi: 10.1007/s11657-016-0262-7 [published Online First: 2016/02/06]  96. Gasparini R, Amicizia D, Lai PL, et al. Effectiveness of adjuvanted seasonal influenza vaccines (Inflexal V (R) and Fluad (R) ) in preventing hospitalization for influenza and pneumonia in the elderly: a matched case-control study. *Hum Vaccin Immunother* 2013;9(1):144-52. doi: 10.4161/hv.22231 [published Online First: 2012/11/13]  97. Townsend LA, Roubion RC, Bourgeois DM, et al. Impact of Age on Patient-Reported Outcome Measures in Total Knee Arthroplasty. *J Knee Surg* 2018;31(6):580-84. doi: 10.1055/s-0037-1605557 [published Online First: 2017/08/26]  98. McCalden RW, Robert CE, Howard JL, et al. Comparison of outcomes and survivorship between patients of different age groups following TKA. *J Arthroplasty* 2013;28(8 Suppl):83-6. doi: 10.1016/j.arth.2013.03.034 [published Online First: 2013/07/31]  99. Song SJ, Bae DK, Kim KI, et al. Long-term survival is similar between closed-wedge high tibial osteotomy and unicompartmental knee arthroplasty in patients with similar demographics. *Knee Surg Sports Traumatol Arthrosc* 2019;27(4):1310-19. doi: 10.1007/s00167-019-05390-w [published Online First: 2019/02/06]  100. Krych AJ, Reardon P, Sousa P, et al. Unicompartmental Knee Arthroplasty Provides Higher Activity and Durability Than Valgus-Producing Proximal Tibial Osteotomy at 5 to 7 Years. *J Bone Joint Surg Am* 2017;99(2):113-22. doi: 10.2106/JBJS.15.01031 [published Online First: 2017/01/19]  101. Karamitev SS, Stavrev VP, Chifligarov AG. Comparative analysis of the results obtained after unicondylar knee arthroplasty and high tibial osteotomy in isolated gonarthrosis. *Folia Med (Plovdiv)* 2014;56(1):11-9. doi: 10.2478/folmed-2014-0002 [published Online First: 2014/05/13]  102. Takeuchi R, Umemoto Y, Aratake M, et al. A mid term comparison of open wedge high tibial osteotomy vs unicompartmental knee arthroplasty for medial compartment osteoarthritis of the knee. *J Orthop Surg Res* 2010;5(1):65. doi: 10.1186/1749-799X-5-65 [published Online First: 2010/08/31]  103. Jeon YS, Ahn CH, Kim MK. Comparison of HTO with articular cartilage surgery and UKA in unicompartmental OA. *J Orthop Surg (Hong Kong)* 2017;25(1):2309499016684092. doi: 10.1177/2309499016684092 [published Online First: 2017/02/09]  104. Petersen W, Metzlaff S. Open wedge high tibial osteotomy (HTO) versus mobile bearing unicondylar medial joint replacement: five years results. *Arch Orthop Trauma Surg* 2016;136(7):983-9. doi: 10.1007/s00402-016-2465-1 [published Online First: 2016/05/08]  105. Tuncay I, Bilsel K, Elmadag M, et al. Evaluation of mobile bearing unicompartmental knee arthroplasty, opening wedge, and dome-type high tibial osteotomies for knee arthritis. *Acta Orthop Traumatol Turc* 2015;49(3):280-7. doi: 10.3944/AOTT.2015.14.0320 [published Online First: 2015/07/23]  106. Yim JH, Song EK, Seo HY, et al. Comparison of high tibial osteotomy and unicompartmental knee arthroplasty at a minimum follow-up of 3 years. *J Arthroplasty* 2013;28(2):243-7. doi: 10.1016/j.arth.2012.06.011 [published Online First: 2012/08/03]  107. Kocak FA, Kurt EE, Milletli Sezgin F, et al. The effect of balneotherapy on body mass index, adipokine levels, sleep disturbances, and quality of life of women with morbid obesity. *Int J Biometeorol* 2020;64(9):1463-72. doi: 10.1007/s00484-020-01924-x [published Online First: 2020/05/08]  108. Beltrame A, Di Benedetto P, Cicuto C, et al. Onlay versus Inlay humeral steam in Reverse Shoulder Arthroplasty (RSA): clinical and biomechanical study. *Acta Biomed* 2019;90(12-S):54-63. doi: 10.23750/abm.v90i12-S.8983 [published Online First: 2019/12/11]  109. Boutsiadis A, Lenoir H, Denard PJ, et al. The lateralization and distalization shoulder angles are important determinants of clinical outcomes in reverse shoulder arthroplasty. *J Shoulder Elbow Surg* 2018;27(7):1226-34. doi: 10.1016/j.jse.2018.02.036 [published Online First: 2018/04/01]  110. Lindbloom BJ, Christmas KN, Downes K, et al. Is there a relationship between preoperative diagnosis and clinical outcomes in reverse shoulder arthroplasty? An experience in 699 shoulders. *J Shoulder Elbow Surg* 2019;28(6S):S110-S17. doi: 10.1016/j.jse.2019.04.007 [published Online First: 2019/06/15]  111. Streit JJ, Shishani Y, Gobezie R. Medialized Versus Lateralized Center of Rotation in Reverse Shoulder Arthroplasty. *Orthopedics* 2015;38(12):e1098-103. doi: 10.3928/01477447-20151120-06 [published Online First: 2015/12/15]  112. Dai WL, Zhang H, Lin ZM, et al. Efficacy of platelet-rich plasma in arthroscopic repair for discoid lateral meniscus tears. *BMC Musculoskelet Disord* 2019;20(1):113. doi: 10.1186/s12891-019-2500-9 [published Online First: 2019/03/20]  113. Everhart JS, Cavendish PA, Eikenberry A, et al. Platelet-Rich Plasma Reduces Failure Risk for Isolated Meniscal Repairs but Provides No Benefit for Meniscal Repairs With Anterior Cruciate Ligament Reconstruction. *Am J Sports Med* 2019;47(8):1789-96. doi: 10.1177/0363546519852616 [published Online First: 2019/06/06]  114. Griffin JW, Hadeed MM, Werner BC, et al. Platelet-rich plasma in meniscal repair: does augmentation improve surgical outcomes? *Clin Orthop Relat Res* 2015;473(5):1665-72. doi: 10.1007/s11999-015-4170-8 [published Online First: 2015/02/11]  115. Pujol N, Salle De Chou E, Boisrenoult P, et al. Platelet-rich plasma for open meniscal repair in young patients: any benefit? *Knee Surg Sports Traumatol Arthrosc* 2015;23(1):51-8. doi: 10.1007/s00167-014-3417-3 [published Online First: 2014/11/08]  116. Bella A, Gesualdo F, Orsi A, et al. Effectiveness of the trivalent MF59 adjuvated influenza vaccine in preventing hospitalization due to influenza B and A(H1N1)pdm09 viruses in the elderly in Italy, 2017 - 2018 season. *Expert Rev Vaccines* 2019;18(6):671-79. doi: 10.1080/14760584.2019.1627206 [published Online First: 2019/06/05]  117. Bellino S, Bella A, Puzelli S, et al. Moderate influenza vaccine effectiveness against A(H1N1)pdm09 virus, and low effectiveness against A(H3N2) subtype, 2018/19 season in Italy. *Expert Rev Vaccines* 2019;18(11):1201-09. doi: 10.1080/14760584.2019.1688151 [published Online First: 2019/11/02]  118. Gilca R, Skowronski DM, Douville-Fradet M, et al. Mid-Season Estimates of Influenza Vaccine Effectiveness against Influenza A(H3N2) Hospitalization in the Elderly in Quebec, Canada, January 2015. *Plos One* 2015;10(7):e0132195. doi: 10.1371/journal.pone.0132195 [published Online First: 2015/07/23]  119. Pebody R, Whitaker H, Zhao H, et al. Protection provided by influenza vaccine against influenza-related hospitalisation in >/=65 year olds: Early experience of introduction of a newly licensed adjuvanted vaccine in England in 2018/19. *Vaccine* 2020;38(2):173-79. doi: 10.1016/j.vaccine.2019.10.032 [published Online First: 2019/10/28]  120. Pebody RG, Whitaker H, Ellis J, et al. End of season influenza vaccine effectiveness in primary care in adults and children in the United Kingdom in 2018/19. *Vaccine* 2020;38(3):489-97. doi: 10.1016/j.vaccine.2019.10.071 [published Online First: 2019/11/07]  121. Puig-Barbera J, Diez-Domingo J, Perez Hoyos S, et al. Effectiveness of the MF59-adjuvanted influenza vaccine in preventing emergency admissions for pneumonia in the elderly over 64 years of age. *Vaccine* 2004;23(3):283-9. doi: 10.1016/j.vaccine.2004.07.017 [published Online First: 2004/11/09]  122. Puig-Barbera J, Diez-Domingo J, Varea AB, et al. Effectiveness of MF59-adjuvanted subunit influenza vaccine in preventing hospitalisations for cardiovascular disease, cerebrovascular disease and pneumonia in the elderly. *Vaccine* 2007;25(42):7313-21. doi: 10.1016/j.vaccine.2007.08.039 [published Online First: 2007/09/25]  123. Spadea A, Unim B, Colamesta V, et al. Is the adjuvanted influenza vaccine more effective than the trivalent inactivated vaccine in the elderly population? Results of a case-control study. *Vaccine* 2014;32(41):5290-4. doi: 10.1016/j.vaccine.2014.07.077 [published Online First: 2014/08/05]  124. Van Buynder PG, Konrad S, Van Buynder JL, et al. The comparative effectiveness of adjuvanted and unadjuvanted trivalent inactivated influenza vaccine (TIV) in the elderly. *Vaccine* 2013;31(51):6122-8. doi: 10.1016/j.vaccine.2013.07.059 [published Online First: 2013/08/13]  125. Izurieta HS, Chillarige Y, Kelman J, et al. Relative Effectiveness of Cell-Cultured and Egg-Based Influenza Vaccines Among Elderly Persons in the United States, 2017-2018. *J Infect Dis* 2019;220(8):1255-64. doi: 10.1093/infdis/jiy716 [published Online First: 2018/12/19]  126. Mannino S, Villa M, Apolone G, et al. Effectiveness of adjuvanted influenza vaccination in elderly subjects in northern Italy. *Am J Epidemiol* 2012;176(6):527-33. doi: 10.1093/aje/kws313 [published Online First: 2012/09/04]  127. March-Lopez P, Madridejos R, Tomas R, et al. Impact of a Multifaceted Antimicrobial Stewardship Intervention in a Primary Health Care Area: A Quasi-Experimental Study. *Front Pharmacol* 2020;11:398. doi: 10.3389/fphar.2020.00398 [published Online First: 2020/04/18]  128. Cies JJ, Moore WS, 2nd, Enache A, et al. beta-lactam Therapeutic Drug Management in the PICU. *Crit Care Med* 2018;46(2):272-79. doi: 10.1097/CCM.0000000000002817 [published Online First: 2017/11/08]  129. Machado AS, Oliveira MS, Sanches C, et al. Clinical Outcome and Antimicrobial Therapeutic Drug Monitoring for the Treatment of Infections in Acute Burn Patients. *Clin Ther* 2017;39(8):1649-57 e3. doi: 10.1016/j.clinthera.2017.06.008 [published Online First: 2017/07/15]  130. Economou CJP, Wong G, McWhinney B, et al. Impact of beta-lactam antibiotic therapeutic drug monitoring on dose adjustments in critically ill patients undergoing continuous renal replacement therapy. *Int J Antimicrob Agents* 2017;49(5):589-94. doi: 10.1016/j.ijantimicag.2017.01.009 [published Online First: 2017/03/28]  131. McDonald C, Cotta MOC, Little PJ, et al. Is high-dose β-lactam therapy associated with excessive drug toxicity in critically ill patients? *Minerva Anestesiologica* 2016;82(9):957-65.  132. Patel BM, Paratz J, See NC, et al. Therapeutic drug monitoring of beta-lactam antibiotics in burns patients--a one-year prospective study. *Ther Drug Monit* 2012;34(2):160-4. doi: 10.1097/FTD.0b013e31824981a6 [published Online First: 2012/03/13]  133. Wong G, Briscoe S, McWhinney B, et al. Therapeutic drug monitoring of beta-lactam antibiotics in the critically ill: direct measurement of unbound drug concentrations to achieve appropriate drug exposures. *J Antimicrob Chemother* 2018;73(11):3087-94. doi: 10.1093/jac/dky314 [published Online First: 2018/08/24]  134. Roberts JA, Ulldemolins M, Roberts MS, et al. Therapeutic drug monitoring of beta-lactams in critically ill patients: proof of concept. *Int J Antimicrob Agents* 2010;36(4):332-9. doi: 10.1016/j.ijantimicag.2010.06.008 [published Online First: 2010/08/06]  135. Schoenenberger-Arnaiz JA, Ahmad-Diaz F, Miralbes-Torner M, et al. Usefulness of therapeutic drug monitoring of piperacillin and meropenem in routine clinical practice: a prospective cohort study in critically ill patients. *Eur J Hosp Pharm* 2020;27(e1):e30-e35. doi: 10.1136/ejhpharm-2018-001713 [published Online First: 2020/04/17]  136. Al-Shaer MH, Rubido E, Cherabuddi K, et al. Early therapeutic monitoring of beta-lactams and associated therapy outcomes in critically ill patients. *J Antimicrob Chemother* 2020;75(12):3644-51. doi: 10.1093/jac/dkaa359 [published Online First: 2020/09/11]  137. Scharf C, Liebchen U, Paal M, et al. The higher the better? Defining the optimal beta-lactam target for critically ill patients to reach infection resolution and improve outcome. *J Intensive Care* 2020;8(1):86. doi: 10.1186/s40560-020-00504-w [published Online First: 2020/12/10]  138. Hwang H, Kim B. Impact of an infectious diseases specialist-led antimicrobial stewardship programmes on antibiotic use and antimicrobial resistance in a large Korean hospital. *Sci Rep* 2018;8(1):14757. doi: 10.1038/s41598-018-33201-8 [published Online First: 2018/10/05]  139. Bishop PA, Isache C, McCarter YS, et al. Clinical impact of a pharmacist-led antimicrobial stewardship initiative evaluating patients with Clostridioides difficile colitis. *J Investig Med* 2020;68(4):888-92. doi: 10.1136/jim-2019-001173 [published Online First: 2020/02/19]  140. Dunn K, O'Reilly A, Silke B, et al. Implementing a pharmacist-led sequential antimicrobial therapy strategy: a controlled before-and-after study. *Int J Clin Pharm* 2011;33(2):208-14. doi: 10.1007/s11096-010-9475-9 [published Online First: 2011/07/12]  141. Pieracci FM, Lin Y, Rodil M, et al. A prospective, controlled clinical evaluation of surgical stabilization of severe rib fractures. *J Trauma Acute Care Surg* 2016;80(2):187-94. doi: 10.1097/TA.0000000000000925 [published Online First: 2015/11/26]  142. Jayle CP, Allain G, Ingrand P, et al. Flail chest in polytraumatized patients: surgical fixation using Stracos reduces ventilator time and hospital stay. *Biomed Res Int* 2015;2015:624723. doi: 10.1155/2015/624723 [published Online First: 2015/02/25]  143. Cai Q, Yang M, Liu D, et al. Experimental Treatment with Favipiravir for COVID-19: An Open-Label Control Study. *Engineering (Beijing)* 2020;6(10):1192-98. doi: 10.1016/j.eng.2020.03.007 [published Online First: 2020/04/30]  144. Abubakar U, Syed Sulaiman SA, Adesiyun AG. Impact of pharmacist-led antibiotic stewardship interventions on compliance with surgical antibiotic prophylaxis in obstetric and gynecologic surgeries in Nigeria. *Plos One* 2019;14(3):e0213395. doi: 10.1371/journal.pone.0213395 [published Online First: 2019/03/08]  145. Wang J, Dong M, Lu Y, et al. Impact of pharmacist interventions on rational prophylactic antibiotic use and cost saving in elective cesarean section. *Int J Clin Pharmacol Ther* 2015;53(8):605-15. doi: 10.5414/CP202334 [published Online First: 2015/06/25]  146. Zhang HX, Li X, Huo HQ, et al. Pharmacist interventions for prophylactic antibiotic use in urological inpatients undergoing clean or clean-contaminated operations in a Chinese hospital. *Plos One* 2014;9(2):e88971. doi: 10.1371/journal.pone.0088971 [published Online First: 2014/03/04]  147. Zhou L, Ma J, Gao J, et al. Optimizing Prophylactic Antibiotic Practice for Cardiothoracic Surgery by Pharmacists' Effects. *Medicine (Baltimore)* 2016;95(9):e2753. doi: 10.1097/MD.0000000000002753 [published Online First: 2016/03/06]  148. Gautret P, Lagier JC, Parola P, et al. Hydroxychloroquine and azithromycin as a treatment of COVID-19: results of an open-label non-randomized clinical trial. *Int J Antimicrob Agents* 2020;56(1):105949. doi: 10.1016/j.ijantimicag.2020.105949 [published Online First: 2020/03/25]  149. Li Z, Cheng B, Zhang K, et al. Pharmacist-driven antimicrobial stewardship in intensive care units in East China: A multicenter prospective cohort study. *Am J Infect Control* 2017;45(9):983-89. doi: 10.1016/j.ajic.2017.02.021 [published Online First: 2017/06/10]  150. Falcone M, Tiseo G, Barbieri G, et al. Role of Low-Molecular-Weight Heparin in Hospitalized Patients With Severe Acute Respiratory Syndrome Coronavirus 2 Pneumonia: A Prospective Observational Study. *Open Forum Infect Dis* 2020;7(12):ofaa563. doi: 10.1093/ofid/ofaa563  10.1093/ofid/ofaa563. eCollection 2020 Dec. [published Online First: 2020/12/29]  151. Geleris J, Sun Y, Platt J, et al. Observational Study of Hydroxychloroquine in Hospitalized Patients with Covid-19. *N Engl J Med* 2020;382(25):2411-18. doi: 10.1056/NEJMoa2012410 [published Online First: 2020/05/08]  152. Magagnoli J, Narendran S, Pereira F, et al. Outcomes of Hydroxychloroquine Usage in United States Veterans Hospitalized with COVID-19. *Med* 2020;1(1):114-27 e3. doi: 10.1016/j.medj.2020.06.001 [published Online First: 2020/08/25]  153. Ip A, Berry DA, Hansen E, et al. Hydroxychloroquine and tocilizumab therapy in COVID-19 patients-An observational study. *Plos One* 2020;15(8):e0237693. doi: 10.1371/journal.pone.0237693 [published Online First: 2020/08/14]  154. Singh S, Khan A, Chowdhry M, et al. Outcomes of hydroxychloroquine treatment among hospitalized COVID-19 patients in the United States—real-world evidence from a federated electronic medical record network. *medRxiv* 2020  155. Mallat J, Hamed F, Balkis M, et al. Hydroxychloroquine is associated with slower viral clearance in clinical COVID-19 patients with mild to moderate disease: a retrospective study. *medRxiv* 2020  156. Park HC, Lee SH, Kim J, et al. Effect of isolation practice on the transmission of middle east respiratory syndrome coronavirus among hemodialysis patients: A 2-year prospective cohort study. *Medicine (Baltimore)* 2020;99(3):e18782. doi: 10.1097/MD.0000000000018782 [published Online First: 2020/02/06]  157. Cochrane E, Pando C, Kirschen GW, et al. Assisted reproductive technologies (ART) and placental abnormalities. *J Perinat Med* 2020;48(8):825-28. doi: 10.1515/jpm-2020-0141 [published Online First: 2020/08/10]  158. Miura M, Ushida T, Imai K, et al. Adverse effects of endometriosis on pregnancy: a case-control study. *BMC Pregnancy Childbirth* 2019;19(1):373. doi: 10.1186/s12884-019-2514-1 [published Online First: 2019/10/24]  159. Berlac JF, Hartwell D, Skovlund CW, et al. Endometriosis increases the risk of obstetrical and neonatal complications. *Acta Obstet Gynecol Scand* 2017;96(6):751-60. doi: 10.1111/aogs.13111 [published Online First: 2017/02/10]  160. Epelboin S, Labrosse J, Fauque P, et al. Endometriosis and assisted reproductive techniques independently related to mother-child morbidities: a French longitudinal national study. *Reprod Biomed Online* 2021;42(3):627-33. doi: 10.1016/j.rbmo.2020.11.017 [published Online First: 2021/01/04]  161. Shmueli A, Salman L, Hiersch L, et al. Obstetrical and neonatal outcomes of pregnancies complicated by endometriosis. *J Matern Fetal Neonatal Med* 2019;32(5):845-50. doi: 10.1080/14767058.2017.1393513 [published Online First: 2017/10/19]  162. Li H, Zhu HL, Chang XH, et al. Effects of Previous Laparoscopic Surgical Diagnosis of Endometriosis on Pregnancy Outcomes. *Chin Med J (Engl)* 2017;130(4):428-33. doi: 10.4103/0366-6999.199840 [published Online First: 2017/02/22]  163. Fujii T, Wada-Hiraike O, Nagamatsu T, et al. Assisted reproductive technology pregnancy complications are significantly associated with endometriosis severity before conception: a retrospective cohort study. *Reprod Biol Endocrinol* 2016;14(1):73. doi: 10.1186/s12958-016-0209-2 [published Online First: 2016/11/05]  164. Benaglia L, Candotti G, Papaleo E, et al. Pregnancy outcome in women with endometriosis achieving pregnancy with IVF. *Hum Reprod* 2016;31(12):2730-36. doi: 10.1093/humrep/dew210 [published Online First: 2016/09/25]  165. Ginstrom Ernstad E, Bergh C, Khatibi A, et al. Neonatal and maternal outcome after blastocyst transfer: a population-based registry study. *Am J Obstet Gynecol* 2016;214(3):378 e1-78 e10. doi: 10.1016/j.ajog.2015.12.040 [published Online First: 2016/03/02]  166. Hassanein M, Abdelgadir E, Bashier A, et al. The role of optimum diabetes care in form of Ramadan focused diabetes education, flash glucose monitoring system and pre-Ramadan dose adjustments in the safety of Ramadan fasting in high risk patients with diabetes. *Diabetes Res Clin Pract* 2019;150:288-95. doi: 10.1016/j.diabres.2018.12.013 [published Online First: 2019/01/15]  167. Ahmedani MY, Alvi SF, Haque MS, et al. Implementation of Ramadan-specific diabetes management recommendations: a multi-centered prospective study from Pakistan. *J Diabetes Metab Disord* 2014;13(1):37. doi: 10.1186/2251-6581-13-37 [published Online First: 2014/02/25]  168. Rebarber A, Istwan NB, Russo-Stieglitz K, et al. Increased incidence of gestational diabetes in women receiving prophylactic 17alpha-hydroxyprogesterone caproate for prevention of recurrent preterm delivery. *Diabetes Care* 2007;30(9):2277-80. doi: 10.2337/dc07-0564 [published Online First: 2007/06/15]  169. Waters TP, Schultz BAH, Mercer BM, et al. Effect of 17alpha-hydroxyprogesterone caproate on glucose intolerance in pregnancy. *Obstet Gynecol* 2009;114(1):45-49. doi: 10.1097/AOG.0b013e3181a9454b [published Online First: 2009/06/24]  170. Wolfe K, Dearmond C, How H, et al. The rates of abnormal glucose challenge tests and gestational diabetes in women receiving 17alpha-hydroxyprogesterone caproate. *Am J Perinatol* 2011;28(10):741-6. doi: 10.1055/s-0031-1280854 [published Online First: 2011/06/11]  171. Nelson DB, McIntire DD, McDonald J, et al. 17-alpha Hydroxyprogesterone caproate did not reduce the rate of recurrent preterm birth in a prospective cohort study. *American Journal of Obstetrics and Gynecology* 2017;216(6):600.e1-00.e9. doi: 10.1016/j.ajog.2017.02.025 |
| --- |
